# Supplementary material for: Evolutionarily distinct lineages of a migratory bird of prey show divergent responses to climate change
Source: Nat Commun. 2025 Apr 13;16:3503. doi: 10.1038/s41467-025-58617-5 (PMC11993763; doi:10.1038/s41467-025-58617-5)
Supplement: Supplementary file 1 — Supplementary Information [file 41467_2025_58617_MOESM1_ESM.pdf]

Supplementary Information for

Evolutionarily distinct lineages of a migratory bird of prey show divergent responses to climate change

Joan Ferrer Obiol[1]*, Anastasios Bounas[2], Mattia Brambilla[1], Gianluca Lombardo[3,4], Simona Secomandi[4,5], Josephine R. Paris[6,7], Alessio Iannucci[8], James R. Whiting[9], Giulio Formenti[10], Andrea Bonisoli-Alquati[11], Gentile Francesco Ficetola[1], Andrea Galimberti[12,13], Jennifer Balacco[10], Nyambayar Batbayar[14], Alexandr E. Bragin[15], Manuela Caprioli[1], Inês Catry[16], Jacopo G. Cecere[17], Batmunkh Davaasuren[14], Federico De Pascalis[17], Ron Efrat[18], Kiraz Erciyas-Yavuz[19], João Gameiro[20,21,22], Gradimir Gradev[23,24], Bettina Haase[10], Todd E. Katzner[25], Jacquelyn Mountcastle[10], Kresimir Mikulic[26], Michelangelo Morganti[13,27], Liviu G. Pârâu[28], Airam Rodríguez[29], Maurizio Sarà[30], Elisavet-Aspasia Toli[2], Nikos Tsiopelas[31], Claudio Ciofi[8], Luca Gianfranceschi[4], Erich D. Jarvis[5,10], Anna Olivieri[3,13], Konstantinos Sotiropoulos[2], Michael Wink[28], Emiliano Trucchi[7], Antonio Torroni[3], Diego Rubolini[1]*

[1]*Dipartimento di Scienze e Politiche Ambientali, Università degli Studi di Milano, Milano, Italy;* [2]*Department of Biological Applications and Technology, University of Ioannina, Ioannina, Greece;* [3]*Dipartimento di Biologia e Biotecnologie "Lazzaro Spallanzani", Università degli Studi di Pavia, Pavia, Italy;* [4]*Dipartimento di Bioscienze, Università degli Studi di Milano, Milan, Italy;* [5]*Laboratory of Neurogenetics of Language, The Rockefeller University, New York, NY, United States;* [6]*Dipartimento di Medicina clinica, Sanità pubblica, Scienze della Vita e dell'Ambiente, Università degli Studi dell'Aquila, Coppito, Italy;* [7]*Dipartimento di Scienze della Vita e dell'Ambiente, Università Politecnica delle Marche, Ancona, Italy*; [8]*Dipartimento di Biologia, Università degli Studi di Firenze, Sesto Fiorentino, Italy;* [9]*Department of Biological Sciences, University of Calgary, Calgary, Alberta, Canada;* [10]*The Vertebrate Genome Laboratory, The Rockefeller University, New York, NY, United States*; [11]*Department of Biological Sciences, California State Polytechnic University - Pomona, Pomona, CA, United States;* [12]*Dipartimento di Biotecnologie e Bioscienze, Università degli Studi di Milano-Bicocca, Milan, Italy;* [13]*National Biodiversity Future Centre (NBFC), Palermo, Italy*; [14]*Wildlife Science and Conservation Center of Mongolia, Ulaanbaatar, Mongolia;* [15]*NGO Naurzum, Kostanay, Kazakhstan;* [16]*Centre for Ecology, Evolution and Environmental Changes (CE3C) & CHANGE – Global Change and Sustainability Institute, Faculdade de Ciências da Universidade de Lisboa, Lisboa, Portugal*; [17]*Area Avifauna Migratrice, Istituto Superiore per la Protezione e la Ricerca Ambientale, Ozzano dell'Emilia, Italy;* [18]*Department of Evolutionary and Environmental Biology, University of Haifa, Haifa, Israel;* [19]*Ornithological Research Center, Ondokuz Mayis University, Samsun, Turkey;* [20]*CIBIO, Centro de Investigação em Biodiversidade e Recursos Genéticos, InBIO Laboratorio Associado, Campus de Vairão, Universidade do Porto, Vairão, Portugal;* [21]*CIBIO, Centro de Investigação em Biodiversidade e Recursos Genéticos, InBIO Laboratorio Associado, Instituto Superior de Agronomia, Universidade de Lisboa, Lisbon, Portugal;* [22]*BIOPOLIS Program in Genomics, Biodiversity and Land Planning, CIBIO, Campus de Vairão, Vairão, Portugal;* [23]*Green Balkans - Stara Zagora NGO, Stara Zagora, Bulgaria;* [24]*Department of Agroecology, Agricultural University - Plovdiv, Plovdiv, Bulgaria;* [25]*U. S. Geological Survey, Boise, ID, USA;* [26]*IBIS program LTD, Zagreb, Croatia;* [27]*Consiglio Nazionale delle Ricerche - Istituto di Ricerca Sulle Acque (CNR-IRSA), Brugherio, Italy;* [28]*Institute of Pharmacy and Molecular Biotechnology, Heidelberg University, Heidelberg, Germany;* [29]*Departamento de Ecología Evolutiva, Museo Nacional de Ciencias Naturales (MNCN), CSIC, Madrid, Spain;* [30]*Dipartimento STEBICEF, Università degli Studi di Palermo, Palermo, Italy;* [31]*Hellenic Ornithological Society, Athens, Greece*

***** *Correspondence to: joan.ferrer.obiol@gmail.com, diego.rubolini@unimi.it*

This file includes:

Supplementary Methods and Results

Supplementary Figures 1-20

Supplementary Tables 1-9

Supplementary References

Any use of trade, firm, or product names is for descriptive purposes only and does not imply endorsement by the U.S. Government.

Supplementary Methods

Supplementary Methods 1: Genome sequencing and assembly

We sampled whole blood from a lesser kestrel trio (both parents and a female offspring) from Matera, Italy and stored it in absolute ethanol (see Supplementary Data 1). DNA extraction was performed according to the Nanobind - Bionano Prep SP Frozen Human Blood DNA Isolation Protocol (Bionano Genomics, Document Number: 30246F) following the manufacturer's protocol. The quality of the extracted ultra-high molecular weight (uHMW) DNA was assessed by pulsed field gel electrophoresis (PFGE; Pippin Pulse, SAGE Science, Beverly, MA), which showed that the length of the isolated DNA was >200 kbp.

To obtain a reference-quality genome we used the Vertebrate Genomes Project (VGP) trio-binning pipeline [1], based on the trio binning approach [2]. Genomic data from four different sequencing technologies were generated for the female offspring sample: Pacific Biosciences (Menlo Park, CA, USA) continuous long reads (CLR) (141.86 Gb; 105×), 10X Genomics (Pleasanton, CA, USA) linked-reads (89.04 Gb; 66×), Bionano Genomics, Inc. (San Diego, CA, USA) DLS optical maps (335×), and Arima Genomics, Inc. (San Diego, CA, USA) v1 Hi-C reads (72.12 Gb; 53×). Additionally, we generated Illumina short reads for the maternal and paternal samples.

Prior to assembly, we estimated genome size, repetitive content and heterozygosity rate using a k-mer based approach implemented in GenomeScope v.2.0 [3], based on a k-mer database from 10X linked reads generated using Meryl v.1.3 [4]. The lesser kestrel genome was assembled on the DNAnexus cloud computing system (https://www.dnanexus.com/). The pipeline consisted of an assembly step, a scaffolding step and a final polishing step. First, the offspring PacBio data were binned into maternal and paternal haplotypes using the parental Illumina reads. The binned PacBio reads were assembled with TrioCanu [2] into phased primary and alternate haplotigs, i.e. haplotype-specific contigs. We polished the primary and alternate phased haplotigs with Arrow (smrtanalysis 5.1.0.26412) using the PacBio reads and we performed a purging step on the primary haplotigs using purge_dups [5] to remove retained alternate haplotigs. We performed three scaffolding steps: (1) two rounds of scaffolding using 10X linked reads with scaff10x v2.0-2.1 (https://github.com/wtsi-hpag/Scaff10X), (2) scaffolding with Bionano optical maps using Bionano Solve v3.2.1; (3) a last scaffolding step using Hi-C reads, implemented in Salsa 2.2 [6]. The final scaffolds were then subjected to a first polishing step with Arrow using the binned PacBio reads and a second step using binned 10X linked reads. The final scaffolds were manually curated using gEVAL [7] and Hi-C contact maps [8]. During curation, the paternal assembly was chosen as the representative one and the W chromosome was added to it from the maternal assembly.

The paternal assembly was functionally annotated using the Eukaryotic Genome Annotation Pipeline v.8 [1,9], and was screened for repetitive elements using a combination of Windowmasker v1.0.1 [10] and RepeatMasker 4.1.0 [11] with Dfam_3.1 [12] (profile HMM library) and Rebase version 20170127 [13]. Functional completeness was evaluated using BUSCO 4.1.4 [14].

Supplementary Methods 2: Genomic data generation

ddRAD sequencing and genotyping - Library preparation of double-digest Restriction-Site Associated DNA (ddRAD) was performed as follows. For each sample, 300 ng of DNA were digested using two restriction enzymes (PstI and EcoRI) in a single reaction. Individually barcoded adapters were ligated onto the digested fragments, which were pooled in multiplexing batches and purified with 1.5 volumes of AMPureXP beads (Agencourt). Pooled libraries were size selected (510-650 bp) using a BluePippin size fractionator (Sage Science, Beverly, MA), and were amplified in 10 PCR cycles using Phusion High-Fidelity PCR Master Mix (New England BioLabs). The libraries were sequenced on an Illumina NovaSeq 6000 instrument (Illumina, San Diego, CA) using 150 bp paired-end sequencing.

We employed two independent methods for SNP calling. The first approach used SAMtools v.1.10 mpileup [15] to pile up the BAM files and BCFtools to call SNPs [16,17]. The second approach used Stacks v.2 [18] to call SNPs using the Bayesian genotype caller [19,20]. The output was filtered for missing data, retaining loci present in all populations (-p 15) and in at least 75% of the individuals within each population (-r 0.75) and to output SNPs in variant call format (VCF) and RADpainter formats. We further removed potential paralogs and sequencing errors through VCFtools v.0.1.16 [21], filtering VCF files to only include biallelic SNPs, to mask genotypes if the per-sample read depth was < 5 (--minDP 5) or the mean per-sample read depth was > 90 (--max-meanDP 90), or if the genotype quality was < 30 (--minGQ 30), and again we filtered for missing data (--max-missing 0.75). We also removed singletons [22] and SNPs located in sex chromosomes. At this point, we intersected the VCF files to obtain a final set of SNPs present in both files.

Mitogenome data generation - To generate mitogenomes, we designed two sets of three primer pairs using Primer3Plus [23] that amplified three overlapping long-range fragments of similar lengths (approx. 6000-6500 bp). The original primer set used the common kestrel (*Falco tinnunculus*) reference mitogenome (NCBI accession: NC_011307) and the second, more specific primer set (Supplementary Table 6) was designed on the obtained lesser kestrel mitogenomes. PCRs were carried out in 50 µl volume with a standard reaction mix containing 1x GoTaq® Long PCR Master Mix (Promega), 0.3 µM of each primer and ~200 ng of DNA template, using the following conditions: 94°C (2 min); 30 cycles at 94°C (30 s), 56°C (30 s), 65°C (7 min) and a final extension at 72°C (10 min). PCR bands were checked by electrophoresis using a 1% agarose gel. PCR purification was performed with Presto™ 96 Well PCR Cleanup Kit (Geneaid). Amplicons were quantified with the Quantus™ fluorometer (Promega) using the QuantiFluor® ONE dsDNA system. For library preparation, the three PCR fragments from each sample were combined in equal concentrations. The sequencing library was prepared with the Nextera™ DNA Flex Library Prep Kit following the manufacturer's protocol. Libraries were then checked on a 2% agarose gel, quantified using a Quantus™ fluorometer (Promega), normalised and pooled together. The pooled normalised library was then run on a 4150 TapeStation System (Agilent) and diluted to 4 nM using RSB resuspension buffer. 5 µl of the pooled library were then denatured using 5 µl of freshly prepared NaOH (0.2N), diluted using HT1 hybridisation buffer (Hyb) to the loading concentration of 6

pM (600 µl final volume), and sequenced on a MiSeq instrument (Illumina) using paired-end sequencing. The reference mitogenome for the species was obtained through the mitoVGP pipeline [24] using the PacBio and 10X linked reads from the female offspring sample.

Supplementary Methods 3: Population structure, gene flow and evolutionarily significant unit (ESU) identification

We first checked the robustness of the ddRAD dataset to detect the primary axis of population differentiation separating the Western and Eastern ESUs. To this end, we randomly downsampled the set of 27,853 SNPs used for this analysis in separate datasets consisting of 20,000, 10,000, 5,000, 2,000 and 500, and then performed a principal component analysis (PCA) for each of the datasets. The results suggest that the power to detect differentiation between the Western and the Eastern ESUs remains the same even when downsampling to 2,000 SNPs. At 500 SNPs, although a reduction in the level of differentiation emerges, PC1 still accounts for 7.23% of the variance and shows some degree of differentiation between the two ESUs.

To investigate the patterns of gene flow among populations across the breeding range, we used estimated effective migration surfaces – EEMS [25]. EEMS uses a stepping-stone model, and - assuming gene flow is symmetric - it assesses whether dispersal rates between adjacent local populations are higher or lower than expected under an isolation-by-distance (IBD) model, returning maps that represent the posterior mean of gene flow illustrating barriers and corridors to gene flow across the landscape. We performed three independent runs consisting of 10,000,000 Markov chain Monte Carlo (MCMC) iterations, discarding the first 1,000,000 iterations as burn-in and sampling every 10,000 iterations in a grid consisting of 200 demes. Results were plotted using the 'reemsplots' R package [25].

We also estimated contemporary gene flow among the four main clusters inferred by fineRADStructure [26] (Iberia, Middle and Eastern Europe, Israel and Asia) using BayesAss3-SNPs [27,28]. BayesAss3 uses a Bayesian approach to infer gene flow rates based on the frequency of individuals with immigrant ancestry [27,28]. Mixing parameters for gene flow rates, allele frequencies and inbreeding coefficients were set to 0.25, 0.4 and 0.03, respectively, after reaching optimal acceptance rates (between 20% and 60%) in a test run. We ran three independent analyses with different random seeds for 5,000,000 iterations, discarding the first 3,000,000 as burn-in and sampling every 100 iterations. We checked for convergence using Tracer v.1.7.1 [29].

To further visualise genealogical patterns, we inferred a Neighbour-net phylogenetic network [30], implemented in SplitsTree5 v.5.0.16 [31]. We first converted the VCF file to a DNAbin object using the vcfR2DNAbin function from the vcfR R package [32] and then computed a genetic distance matrix using the dist.dna function from the ape R package [33], which we used as input for SplitsTree5.

We calculated genome-wide individual heterozygosity for the six individuals with whole-genome sequencing (WGS) data as the number of heterozygous genotypes divided by the total number of callable positions of each individual. To determine the total number of callable positions, we generated a mappability mask using GenMap [34] with a k-mer size of 150 bp and allowing for up to two mismatches.

Sites with a mappability score of < 0.5 were masked and we also masked sites within annotations of repetitive regions (see Supplementary Methods S1). We also applied a per-individual depth filter, set at >⅓× and <2× the average genome-wide depth for each sample to control for differences in coverage between samples. We then calculated heterozygosity in nonoverlapping 1 Mb windows across all autosomes for each individual. We excluded windows where more than 50% of sites were masked.

Supplementary Methods 4: Modelling current, past and future distribution

Separate species distribution models (SDMs) were produced for each combination of ESU (Western, Eastern) and season (breeding, non-breeding). SDMs were based on a large database of breeding and non-breeding records, assembled from different published and unpublished sources (Supplementary Table 7).

Regarding breeding data, based on the population structure analyses (Fig. 2a-d), we considered 1,806 breeding records belonging to the Western ESU (collected between 1978 and 2021, mean year 2018) and 657 belonging to the Eastern one (collected between 1993 and 2021, mean year 2013; Supplementary Table 7). For most European countries hosting large breeding populations (Portugal, Spain, Italy, Greece), we exploited highly accurate national population census data (Supplementary Table 7). For the other regions, most data were obtained from citizen science databases (mainly eBird and GBIF). Citizen science records were carefully screened to remove implausible observations (e.g. those outside the known breeding range, likely reflecting migrating individuals). We also removed citizen science records from those countries/regions for which we had accurate national population census information. As a consequence, from the GBIF.org [35] dataset (11,800 observations of lesser kestrels during May-June, i.e. the core breeding season), we retained 494 records only. For the eBird [36] dataset, we considered only those records with breeding status defined as possible, probable or confirmed (breeding category codes C2, C3 and C4, respectively).

Non-breeding distribution data consisted mainly of citizen science records from GBIF (Supplementary Table 7), with the addition, for the Western ESU, of non-breeding areas of individually tracked birds [37]. A total of 229 and 3,936 records were obtained for the Western and Eastern ESUs, respectively. From the GBIF.org [38] dataset (lesser kestrel observations during December and January), we retained 4,051 records out of 5,160 original ones, excluding records without photographic evidence and those that were outside the core non-breeding areas in sub-Saharan Africa (e.g. a few records of lesser kestrels spending the non-breeding season in Iberia and north Africa). Non-breeding distribution data were collected between 2001 and 2020.

Records within the same 2.5 arc-minute grid cell (corresponding to the spatial resolution of climatic data) and duplicate records were pooled into a single occurrence record before fitting SDMs. Hence, original breeding distribution data were converted into 962 and 360 breeding occurrence records for the Western and Eastern ESUs, while for the non-breeding season the corresponding figures were 159 and 1,691 occurrence records, respectively (Supplementary Data 3) (Fig. 1b; Supplementary Fig. 14).

Considering the global scale of the study, we initially positioned background locations throughout all terrestrial regions included in a rectangular area defined by minimum and maximum latitude and longitude of 2,000 km-buffers centred on all occurrence records (min. longitude 45.60° E, max. longitude 170.19° W, min. latitude 39.64° S, max. latitude 83.60° N). We thus left out of this global background only extremely distant areas, hardly accessible to lesser kestrels. Within that area, we randomly scattered 200,000 background locations (excluding open water). Given that background choice could affect model outcomes, we rerun the analyses using background locations specific to each modelling unit (four combinations of seasons and ESUs). For each modelling unit, we used the global background locations located within a 1,500 km-buffer around occurrence records for that modelling unit, which were the same buffers used for cropping predicted distributions (see main text). We subsequently checked for consistency in the modelled environmental suitability between the models using the global background and the models using the modelling unit specific backgrounds.

Regardless of background choice, for all four models, occurrence data were partitioned into spatially independent training and testing subsets using the "checkerboard 2" method (aggregation factors: 10 and 2) of the R package ENMeval [39], which subdivided data into four partitions. A subset including the data from three partitions was used as a training dataset, whereas data from the fourth partition were used as testing dataset. SDMs were implemented using MaxEnt, a presence-background SDM algorithm which has been previously shown to outperform other methods for occurrence-only data [40–44]. MaxEnt models were built following the procedure outlined in Brambilla et al. [43], aimed at reducing overfitting by using only linear and quadratic features, and selecting and tuning different model parameters based on the Akaike's Information Criterion corrected for small sample size (AICc) to balance power and complexity [45]. The modelling procedure involved the following main steps: selection of the initial regularisation multiplier; removal of variables with $\lambda = 0$; 'final' model tuning, including the selection of the final regularisation multiplier value, fitting function (linear and/or quadratic), iteration number, and climate variables to be included by testing the effect of variable removal, according to the relative value of permutation importance (Supplementary Table 8). None of the four climate variables was removed from the final model (see Brambilla et al. [43] for further details). Model reliability was evaluated on its performance over training and test datasets for each combination of ESU and season. It was assessed by calculating and comparing TSS (True Skill Statistic) as well as AUC (Area Under the Curve of the receiver operating characteristics) over training and test datasets for each combination of ESU and season. The use of such statistics (which are sensitive to prevalence and cannot be used as an absolute measure of model accuracy) over independent datasets offers a way to evaluate model robustness and generalisability. The values of AUC and TSS were always similar between training and test datasets (maximum difference across all combinations < 0.02, except for TSS for the non-breeding distribution model for the Western ESU, which was equal to 0.05). We also calculated the omission rates on test data at two thresholds computed on training data, i.e. the 10th percentile on training data, and the minimum training presence. Omission rates in test data close to expected values of 0.1 and 0, respectively, indicate model robustness; this was verified for all models, with omission rates at 10th percentile always between 0.05 and 0.16, and omission rates at minimum training presence equal to 0.

Results from the models including background locations specific to each modelling unit were highly correlated with those from the models including all background locations. For each modelling unit, the ranking of variables' according to permutation importance was consistent between the two models with different backgrounds. Suitability values at occurrence locations were strongly correlated for non-breeding Western ESU (r = 0.97), breeding Eastern ESU (r = 0.96) and non-breeding Eastern ESU (r = 0.81), and highly correlated for breeding Western ESU (r = 0.57). For the latter, we compared the predicted distribution from the two models with the known distribution of the species: both models depicted a broadly similar distribution of suitable areas, but the one based on the broader background led to a predicted distribution that was more consistent with the actual one. All analyses were therefore based on models using the broader background, which we deemed as more reliable. The models were converted into presence-absence distribution maps using commonly adopted thresholds [43,46]. Upon visually assessing predicted distributions obtained with different thresholds, the best agreement with known distributions was achieved with the following (cloglog) suitability values: 0.4000 for breeding Eastern ESU, 0.2201 for breeding Western ESU, 0.1624 for non-breeding Eastern ESU, 0.1084 for non-breeding Western ESU.

Supplementary Methods 5: Detection of loci associated with climatic variation

Because loadings of sampling localities on RDA1 were very similar to the main axis of population structure and to longitudinal variation, we employed a further strategy to filter out SNPs that were more associated with either population structure or geography than with climate. We did not completely discard SNPs correlated with either population structure or geography (in contrast to e.g. partial RDA, BayPass or LFMM-LEA [47–49]) because in our case climatic variation (and thus potential climate-associated genetic variation) was correlated with population structure (PC1 of bioclimatic variables vs. PC1 of population structure: r = 0.92, p < 0.001) and geography (PC1 of bioclimatic variables vs. longitude: r = 0.88, p < 0.001). Consequently, by discarding SNPs correlated with either population structure or geography, we would be removing the majority of climate-associated genetic variation [50,51]. For each candidate SNP, we modelled the allele frequencies across populations as a function of: (1) the first three PCs of bioclimatic variables (correlation with climate), (2) the first three PCs of population structure (correlation with population structure), and (3) longitude (correlation with geography). For each of the latter, we fitted linear regressions and calculated the R^2. We then kept SNPs that had a higher correlation with climate than with population structure and geography (higher R^2), and had an R^2 with climate > 0.5. When more than one SNP per contig existed, we only retained the SNP showing the highest R^2 in the correlation with climate.

We then performed two differentiation-based analyses using OutFLANK v.0.2 [52] and PCAdapt v.4.3.3 [53]. OutFLANK infers the distribution of F_{ST} for loci that are unlikely to be under selection (based on a trimmed distribution of F_{ST} values) and then attempts to identify outlier loci that have elevated F_{ST} values. Left and right trim fractions were set to 0.35 and 0.06 respectively, and the number of populations was set to K = 2 based on the population structure results. Minimum heterozygosity required

for inclusion was 0.1 and the desired false discovery rate threshold was set to 0.05. PCAdapt detects outlier loci with respect to how they are related to population structure without *a priori* grouping individuals into populations. We retained two principal components based on the inspection of a scree plot and we set an FDR threshold of 0.05 using the R package 'qvalue' v.2.16 [54]. Loci detected as outliers by both OutFLANK and PCAdapt were considered differentiation outliers as they are more likely to be under selection than outlier loci identified by a single method [55].

Supplementary Methods 6: Demographic history reconstruction

To test the robustness of the DIYABC results, we performed the analyses using two different minor allele frequency filters (> 0.01 and > 0.05). As we only aimed at including independent SNPs, we kept a single SNP every 10 kbp (*n* = 7,643 SNPs). For model choice, we simulated a training set of 62,350 data sets (100,000 for parameter estimation) and calculated 130 summary statistics for observed and simulated data. We used five noise variables and generated 500 random forest trees to select the most likely scenario. For parameter estimation of the chosen model, we used 1,000 out-of-bag testing samples. Due to a lack of biological information, we set broad priors drawn from uniform distributions (Supplementary Table 9).

In addition, we used mitogenome coding-regions (15,663 bp) to estimate haplogroup ages using Beast v.2.6.3 [56]. To find the best priors for Bayesian analysis, maximum likelihood (ML) estimations were performed using the BaseML in PAMLX v.1.3.1 [57] assuming a HKY85 mutation model with gamma-distributed rates (approximated by a discrete distribution with 32 categories plus invariant sites [58,59]) and 17 partitions (one rate for each protein-coding gene, two for the rRNA genes, one for all tRNA and one for intergenic regions), using the tree obtained by the Maximum Parsimony approach. We converted ML mutational distances into years by assuming an estimated split time between *F. tinnunculus* and *F. naumanni* of 3.9 Mya (95% CI: 2.7-5.2 Mya) [60]. We then used Beast 2.6.3 with a HKY substitution model (gamma-distributed rates plus invariant sites) and a relaxed clock (log-normal). The clock value derived from BaseML (1.941×10^{-8} base substitution per nucleotide per year) was entered as prior. The chain length was established at 50,000,000 iterations, with samples drawn every 1,000 Markov chain Monte Carlo (MCMC) steps after a discarded burn-in of 5,000,000 steps.

Supplementary Figures

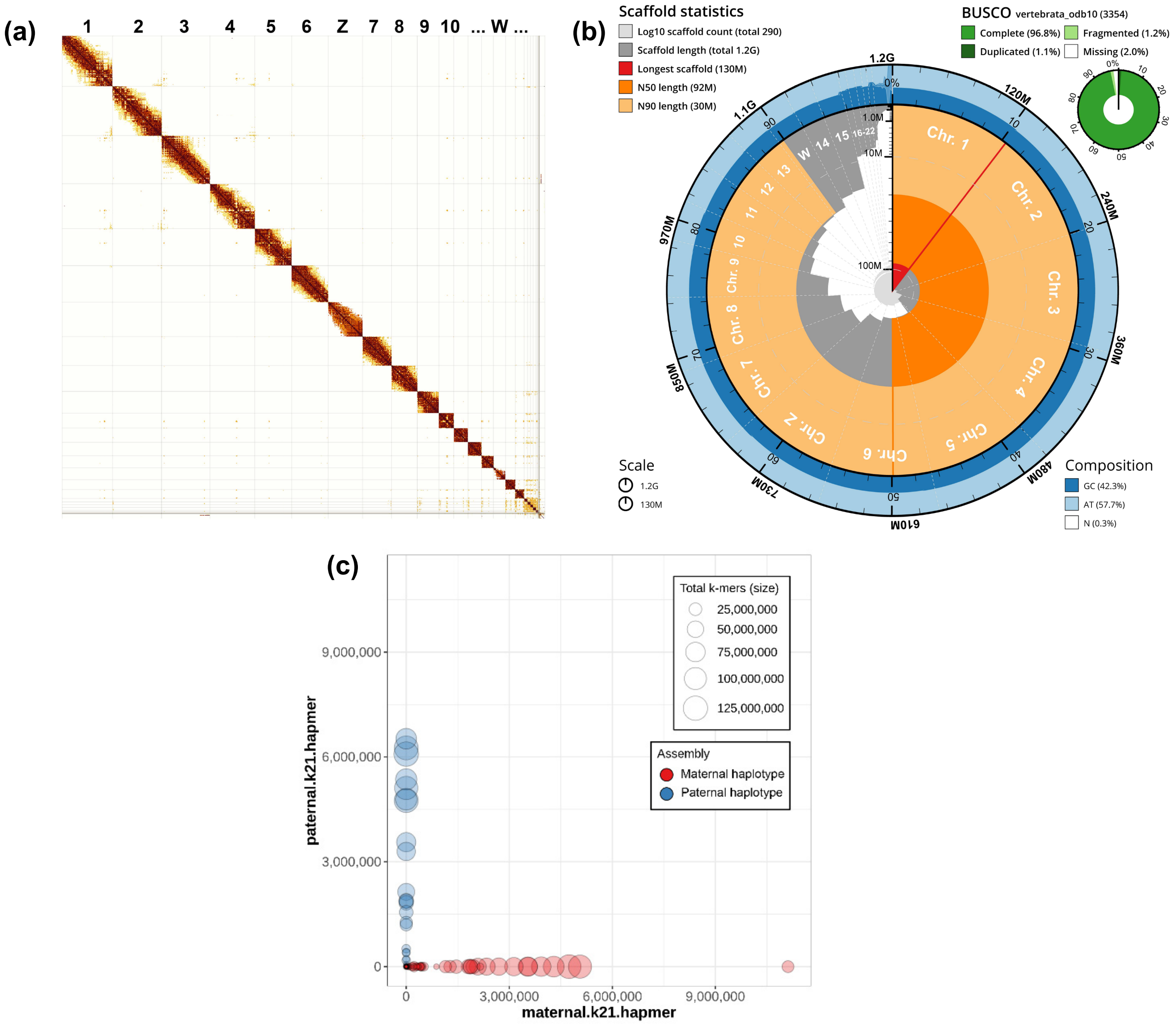

Supplementary Fig. 1 | A chromosome-level genome assembly for the lesser kestrel. **a** Hi-C interaction heatmap for the assembly after manual curation. Both axes represent the linear sequence of the reference genome and the frequency at which two DNA fragments physically associate in 3D space is shown by the colour intensity, with more intense colours representing a higher physical association. **b** Snail plot summary of assembly statistics. The main plot is divided into 1,000 size-ordered bins around the circumference with each bin representing 0.1% of the 1.22 Gbp assembly. The distribution of scaffold lengths is shown in dark grey with the plot radius scaled to the longest scaffold present in the assembly (127 Mbp, in red). Orange and pale-orange arcs show the N50 and N90 scaffold lengths (92 Mbp and 30 Mbp), respectively. The pale grey spiral shows the cumulative scaffold count on a log scale with white scale lines showing successive orders of magnitude. The blue and pale-blue areas around the outside of the plot show the distribution of GC, AT and N percentages in the same bins as the inner plot. A summary of complete, fragmented, duplicated and missing BUSCO genes in the vertebrata_odb10 database is shown in the top-right. **c** Hap-mer blob plot of the lesser kestrel assembly. Each blob represents a contig, and its size is proportional to contig size. Red blobs represent maternal haplotype contigs, whereas blue blobs represent paternal haplotype contigs. Each blob is plotted according to the number of paternal and maternal hap-mers that it contains. The lack of paternal-specific hap-mers in the maternal assembly and *vice versa* suggests that each contig was successfully phased.

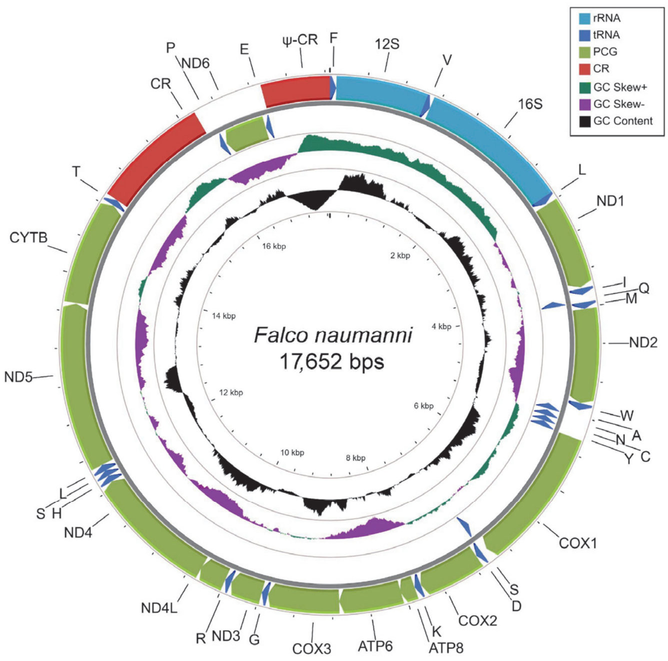

Supplementary Fig. 2 | Graphical representation of the complete lesser kestrel mitogenome. The mitogenome is represented by the grey ring. Genes encoded by the + strand are shown outside the grey ring, and those encoded by the - strand inside the grey ring. Inner rings show GC content and GC skewness.

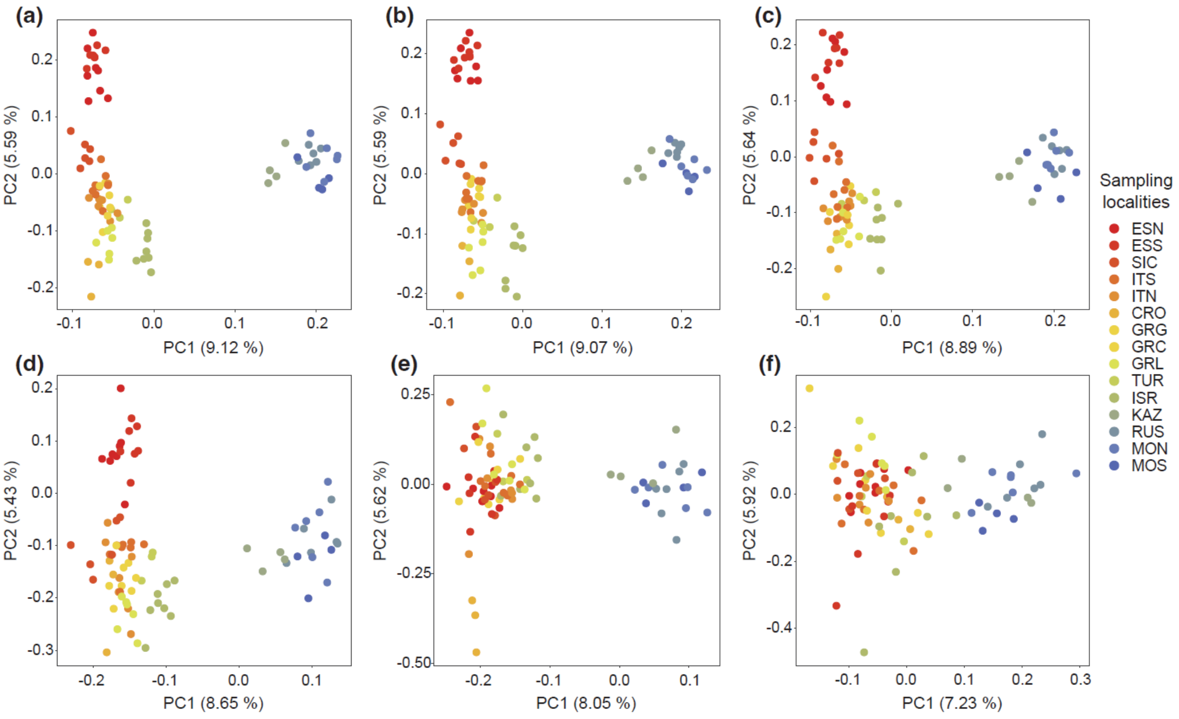

Supplementary Fig. 3 | Downsampling of loci does not affect patterns of population genetic structure in lesser kestrels. Test performed to assess how the number of single-nucleotide polymorphisms (SNPs) from double-digest Restriction-Site Associated DNA (ddRAD) data influenced the ability of Principal Component Analysis (PCA) to detect the primary axis of population differentiation, which separates the Western and Eastern evolutionarily significant units (ESUs). Panels show PCAs using **(a)** the complete linkage-pruned dataset comprising 27,853 SNPs; **(b)** 20,000 SNPs; **(c)** 10,000 SNPs; **(d)** 5,000 SNPs; **(e)** 2,000 SNPs; **(f)** 500 SNPs. Abbreviations for sampling localities are defined in Supplementary Table 1. Data are provided at https://doi.org/10.5281/zenodo.14988067.

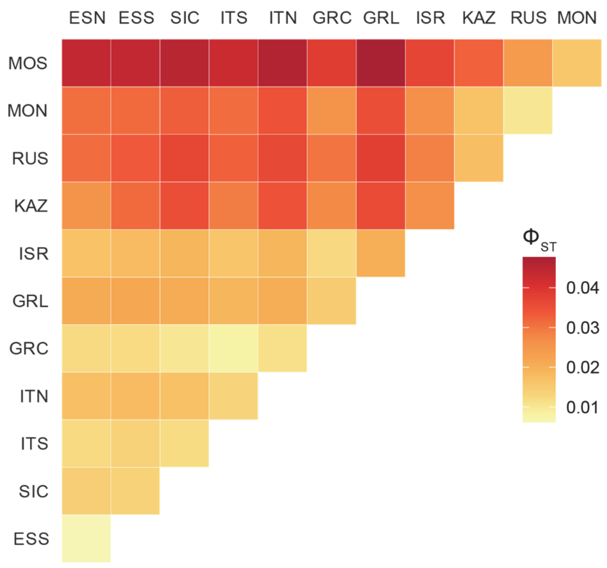

Supplementary Fig. 4 | Heatmap of pairwise Φ_{ST} among sampling localities across the lesser kestrel breeding range. Abbreviations are defined in Supplementary Table 1. Data are provided at https://doi.org/10.5281/zenodo.14988067.

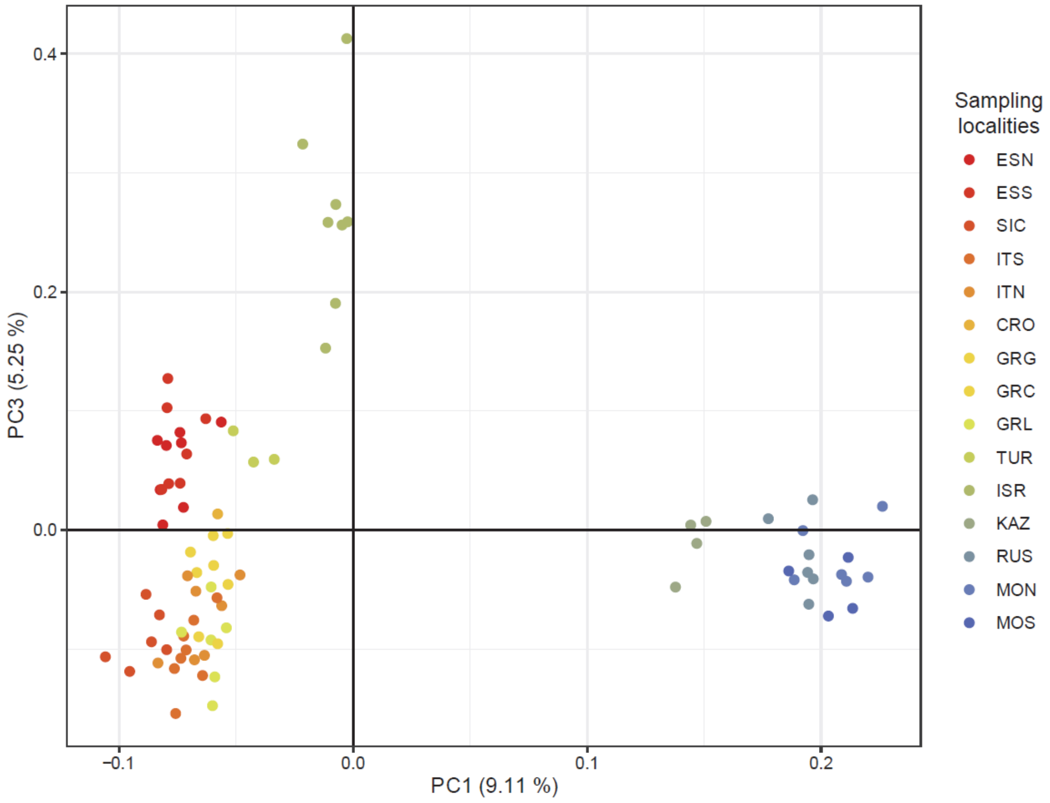

Supplementary Fig. 5 | Principal component analysis (PCA) based on lesser kestrel double-digest Restriction-Site Associated DNA (ddRAD). Principal component 1 (PC1) and principal component 3 (PC3), which separates individuals from Israel from the rest of individuals from the Western evolutionarily significant unit (ESU), are reported. Abbreviations for sampling localities are defined in Supplementary Table 1. Data are provided at https://doi.org/10.5281/zenodo.14988067.

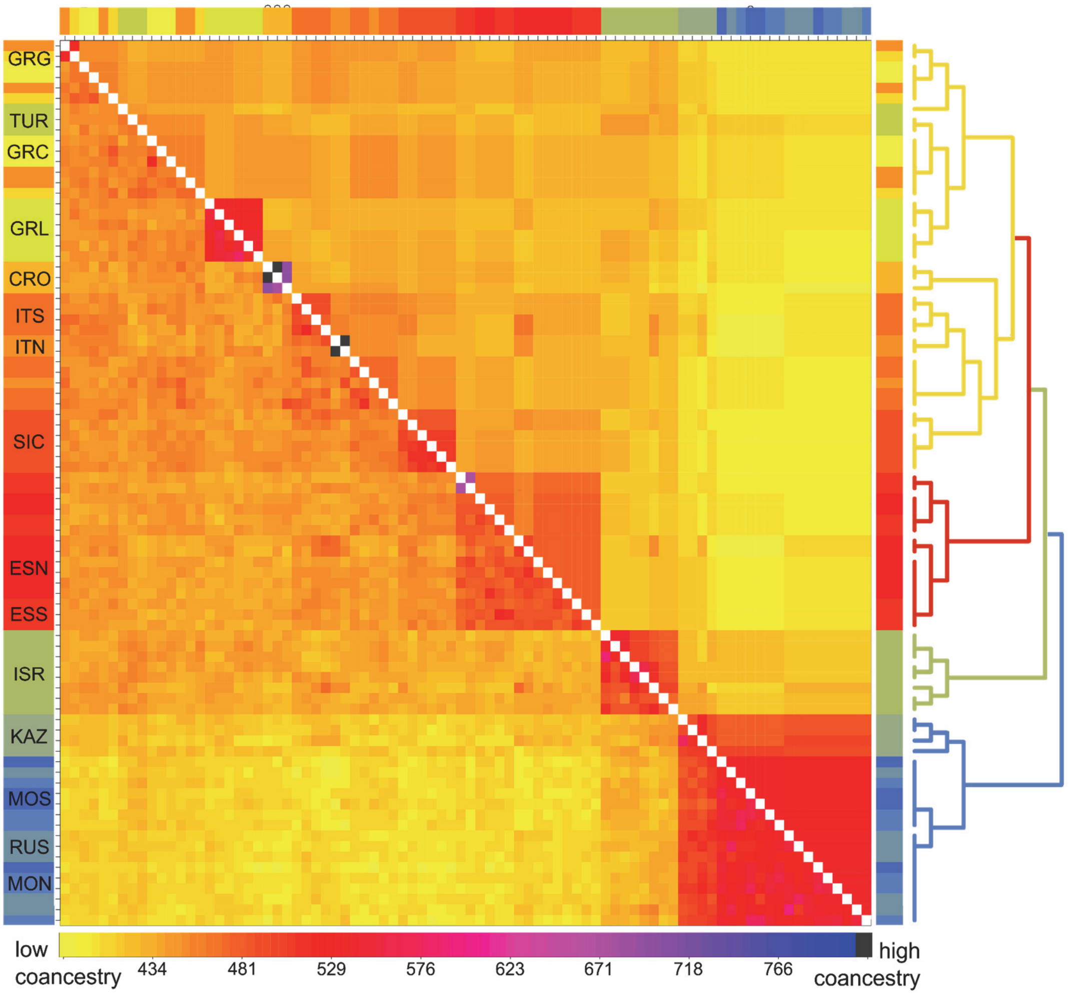

Supplementary Fig. 6 | Coancestry matrix of lesser kestrel individuals across the global distribution range. Pairwise coancestry coefficients based on double-digest Restriction-Site Associated DNA (ddRAD) data, colour-coded from low (yellow) to high (black), and clustering dendrogram based on the coancestry matrix, with branches coloured according to the four fine-scale genetic clusters (Iberian peninsula in red, Italian and Balkan peninsulas – including Turkey – in yellow, Israel in green and Asia in blue) identified by the FineRADStructure structure analysis. Coloured bars at the left, top and right of the coancestry matrix represent sampling localities of each individual (see Supplementary Table 1 for the explanation of abbreviations). Data are provided at https://doi.org/10.5281/zenodo.14988067.

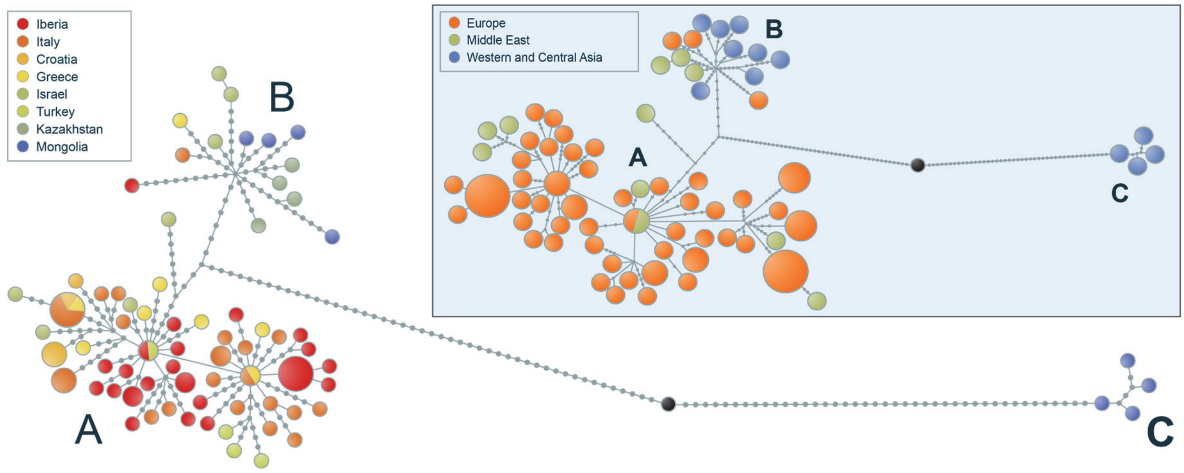

Supplementary Fig. 7 | Haplotype genealogy graph of lesser kestrel mitogenome variation. A, B and C indicate haplogroup affiliations. Node sizes are proportional to haplotype frequencies and are coloured by sampling locality in the main plot and by geographical region in the inset. The inset is drawn collapsing haplotypes separated by less than one substitution (–e 1 option).

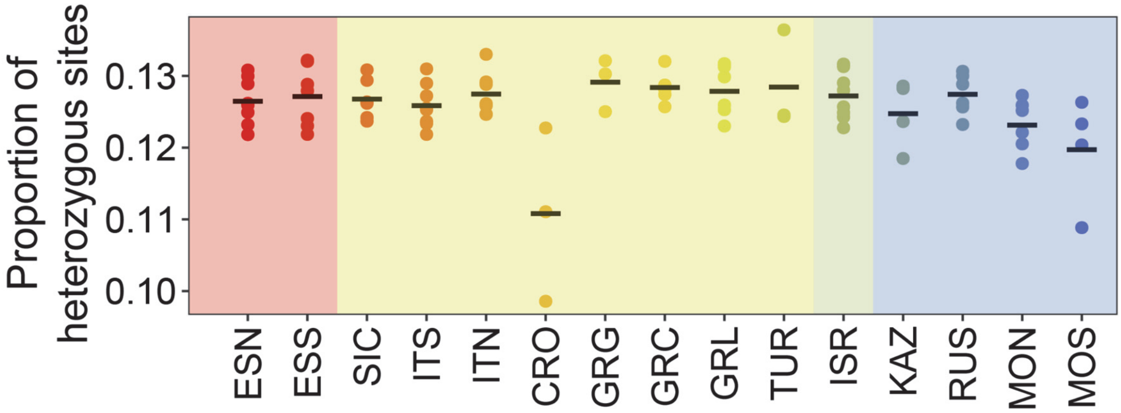

Supplementary Fig. 8 | Variation in individual heterozygosity among lesser kestrel sampling localities. Individual heterozygosity was expressed as the proportion of heterozygous sites per individual for each sampling locality (see Supplementary Table 1 for the explanation of abbreviations), with mean values shown as horizontal black lines. The background is coloured according to the four fine-scale genetic clusters identified by the fineRADStructure analysis (Supplementary Fig. 6). Data are provided at https://doi.org/10.5281/zenodo.14988067.

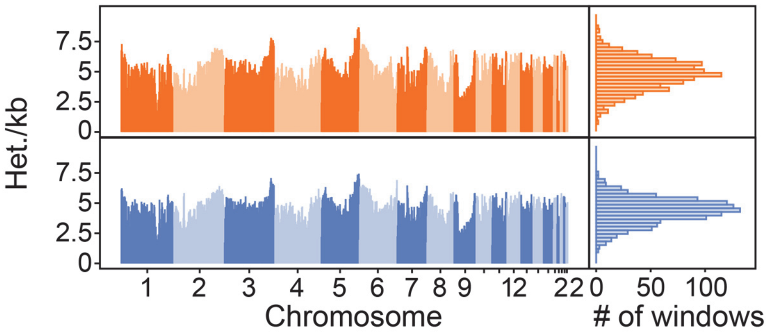

Supplementary Fig. 9 | Variation in heterozygosity across the lesser kestrel genome. Mean individual heterozygosity expressed as the number of heterozygous single-nucleotide polymorphisms (SNPs) per Kb in nonoverlapping 1-Mb windows across the autosomal genome using whole-genome sequencing (WGS) data for Western (top panel) and Eastern (bottom panel) evolutionarily significant units (ESUs) (left); frequency distribution of per-window heterozygosity (right). Data are provided at https://doi.org/10.5281/zenodo.14988067.

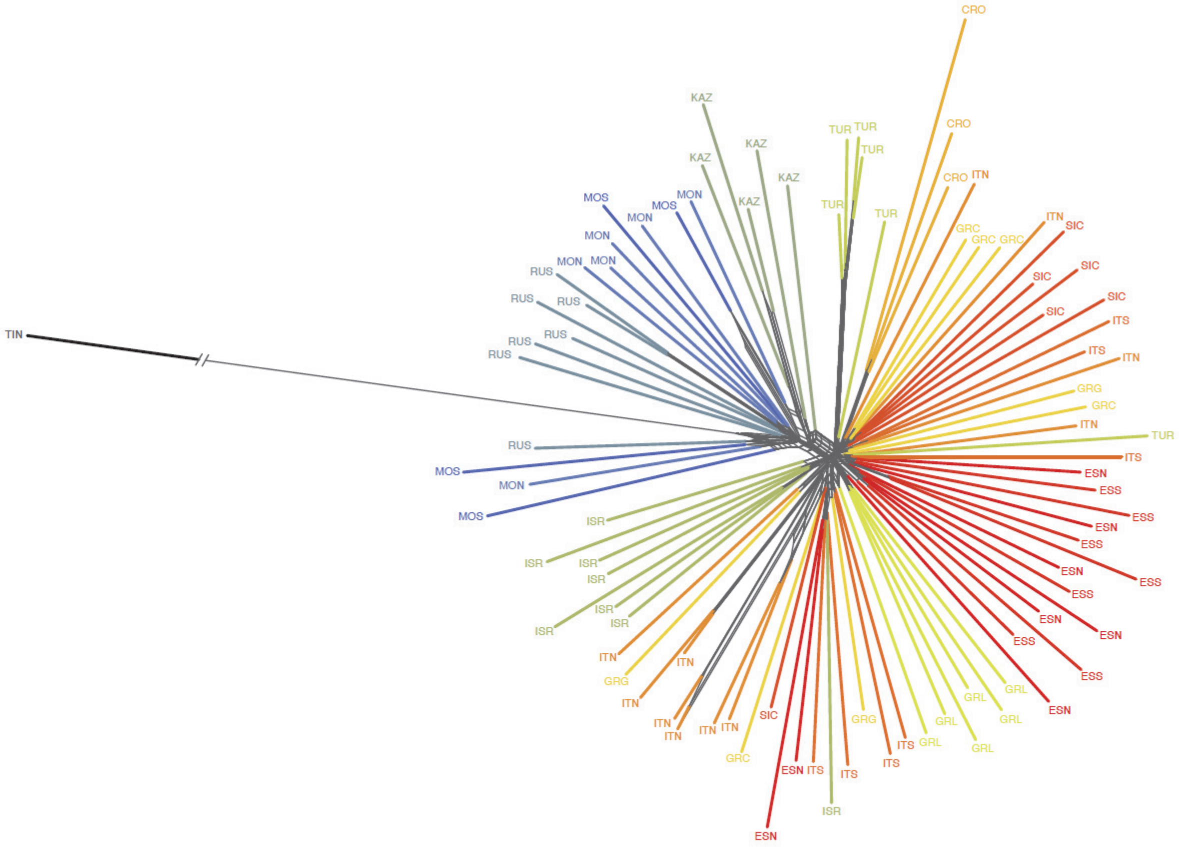

Supplementary Fig. 10 | Neighbour-net network supporting the separation of lesser kestrel Western and Eastern evolutionarily significant units (ESUs). The nearly star-like shape is consistent with the low differentiation among populations. The outgroup branch (*Falco tinnunculus*, TIN) roots the network within the Eastern ESU, which is paraphyletic and suggests an Asian origin for the species. Branches are coloured by sampling locality and abbreviations are defined in Supplementary Table 1. Data are provided at https://doi.org/10.5281/zenodo.14988067.

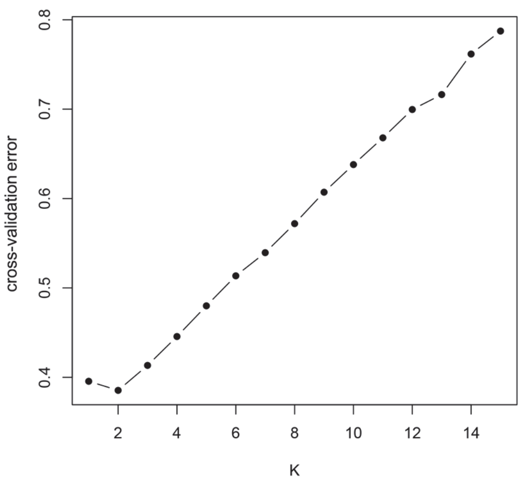

Supplementary Fig. 11 | Admixture cross-validation plot for lesser kestrel double-digest Restriction-Site Associated DNA (ddRAD) data. The plot highlights that *K* = 2 is the best supported number of clusters based on cross-validation. Data are provided at https://doi.org/10.5281/zenodo.14988067.

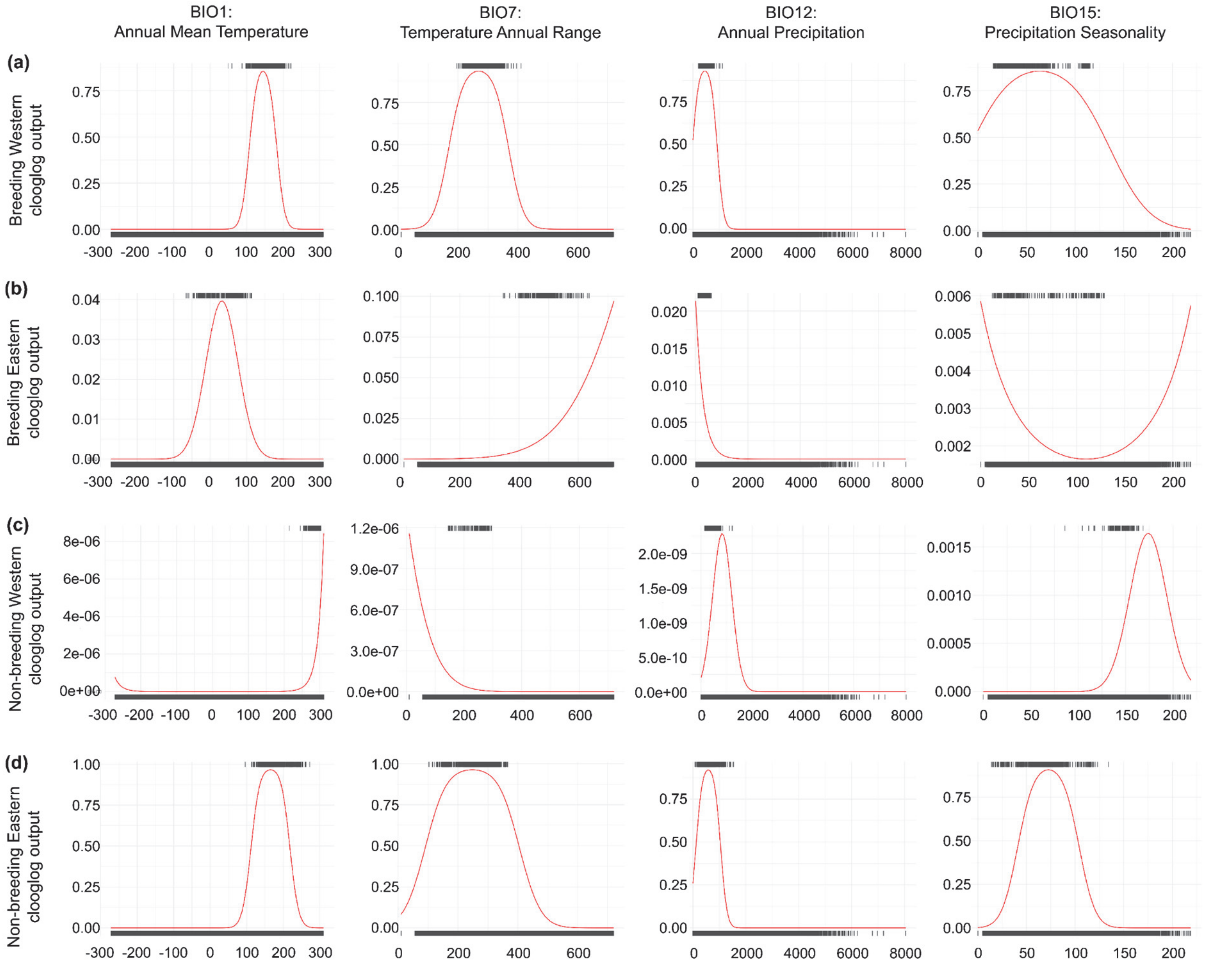

Supplementary Fig. 12 | Response curves to four bioclimatic variables for lesser kestrel species distribution models (SDMs). Models were built for **(a)** the Western ESU during the breeding season, **(b)** the Eastern ESU during the breeding season, **(c)** the Western ESU during the non-breeding season, and **(d)** the Eastern ESU during the non-breeding season. BIO1 (annual mean temperature) and BIO7 (temperature annual range) are expressed in °C × 10; BIO12 (annual precipitation) and BIO15 (precipitation seasonality) in kg/m^2 per year.

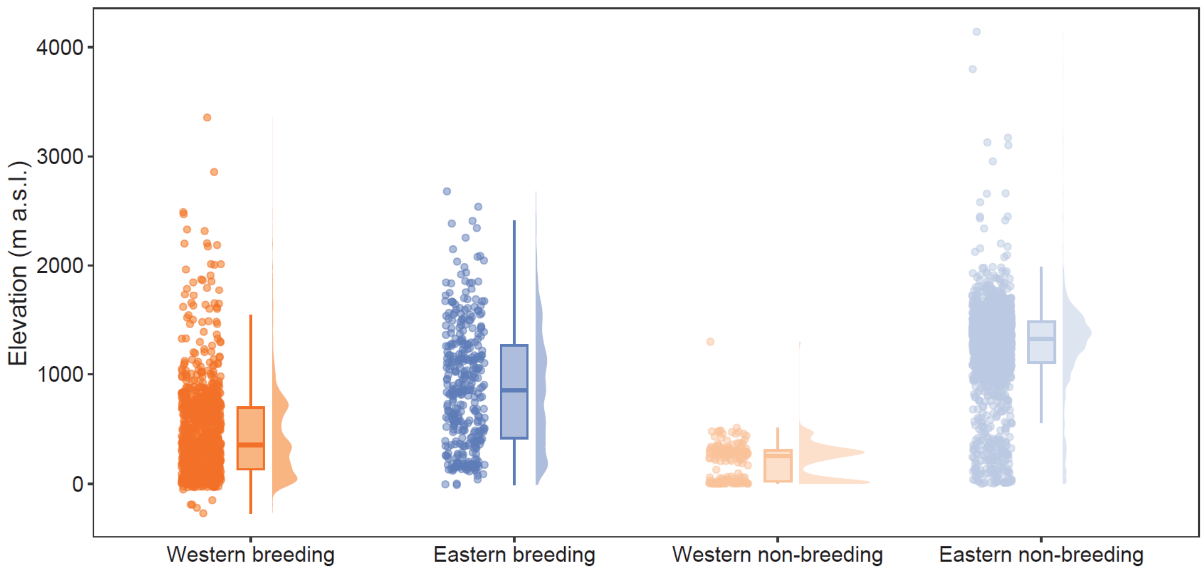

Supplementary Fig. 13 | Elevational distribution of lesser kestrels during the breeding and non-breeding seasons. Raincloud plots of elevation (m a.s.l.) of the grid cells (100 m × 100 m) with lesser kestrel occurrence in the two evolutionarily significant units (ESUs) during the breeding and non-breeding seasons. Data are provided at https://doi.org/10.5281/zenodo.14988067.

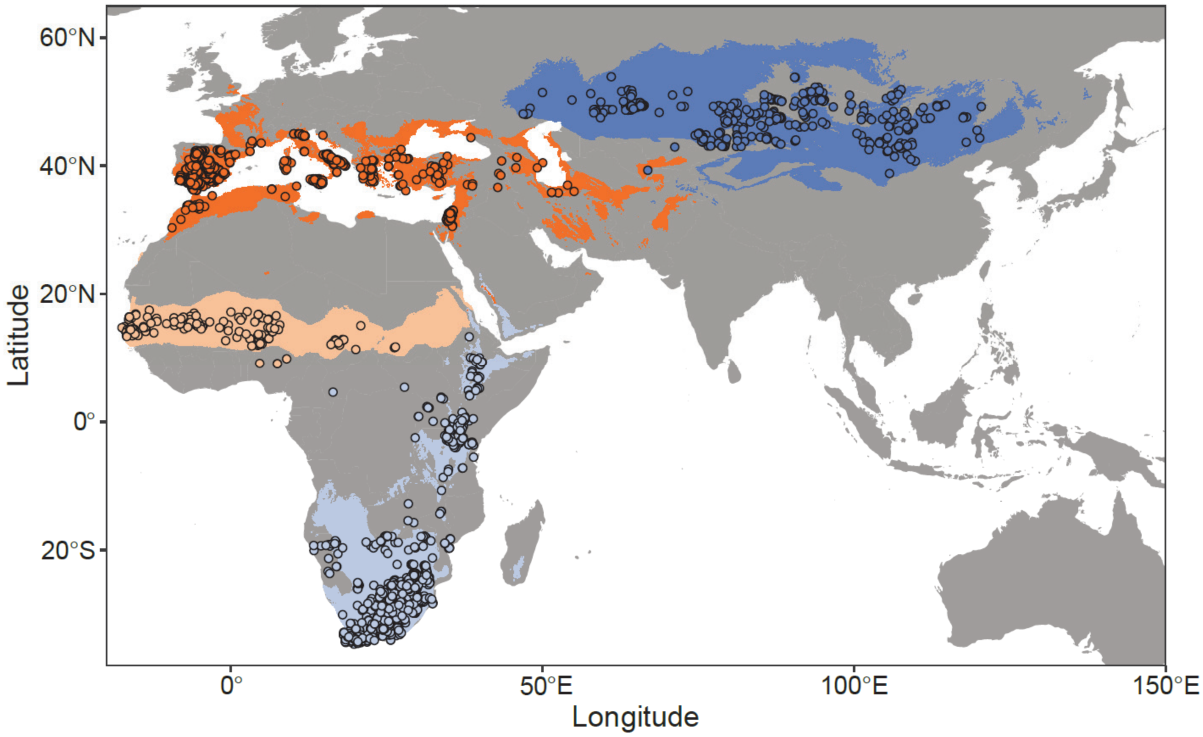

Supplementary Fig. 14 | Lesser kestrel occurrence records and predicted distribution ranges. Dots show the original occurrence data (pooled for 2.5 arc-minute grid cells) used for species distribution modelling together with the predicted current breeding (dark-shaded colours) and non-breeding (light-shaded colours) distribution ranges for Western (orange) and Eastern (blue) evolutionarily significant units (ESUs) based on bioclimatic variables. Data are provided at https://doi.org/10.5281/zenodo.14988067. Background maps were obtained from the rnaturalearth v.0.3.2 R package.

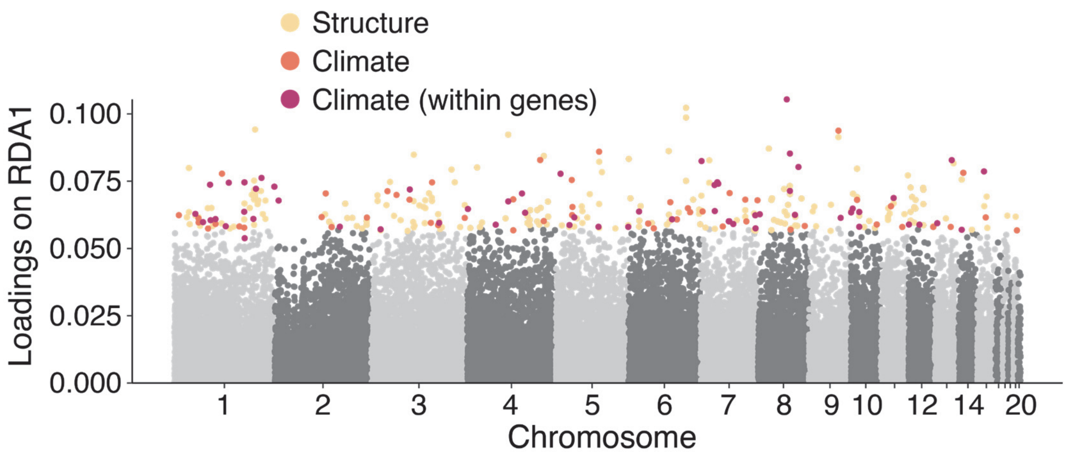

Supplementary Fig. 15 | Manhattan plot showing lesser kestrel single-nucleotide polymorphism (SNP) associations with the first redundancy analysis (RDA) axis. The climate-associated candidate SNPs are coloured in dark red (within genes) and orange (outside genes), and SNPs more associated with population structure are coloured in yellow. Chromosomes are coloured in alternating light and dark grey. Data are provided at https://doi.org/10.5281/zenodo.14988067.

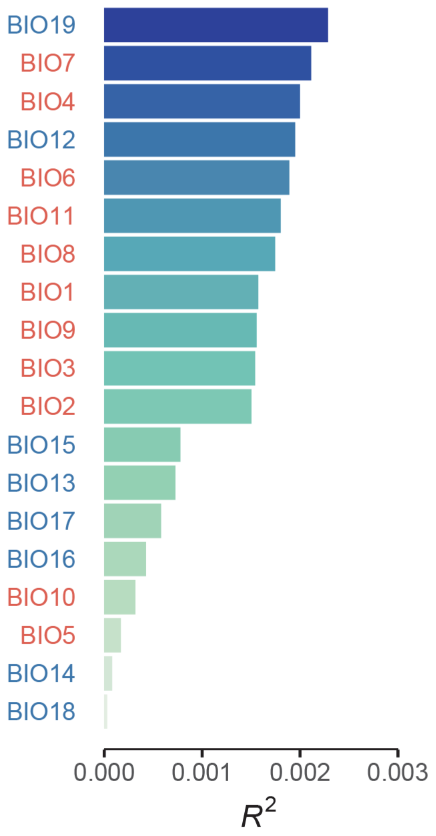

Supplementary Fig. 16 | Relative importance (R^2) of bioclimatic variables in explaining variation in allele frequencies at 61 climate-associated SNPs according to a gradient forest (GF) analysis. Bioclimatic variables associated with temperature are shown in red and those associated with precipitation are shown in blue (see Supplementary Table 5 for details of bioclimatic variables). Data are provided at https://doi.org/10.5281/zenodo.14988067.

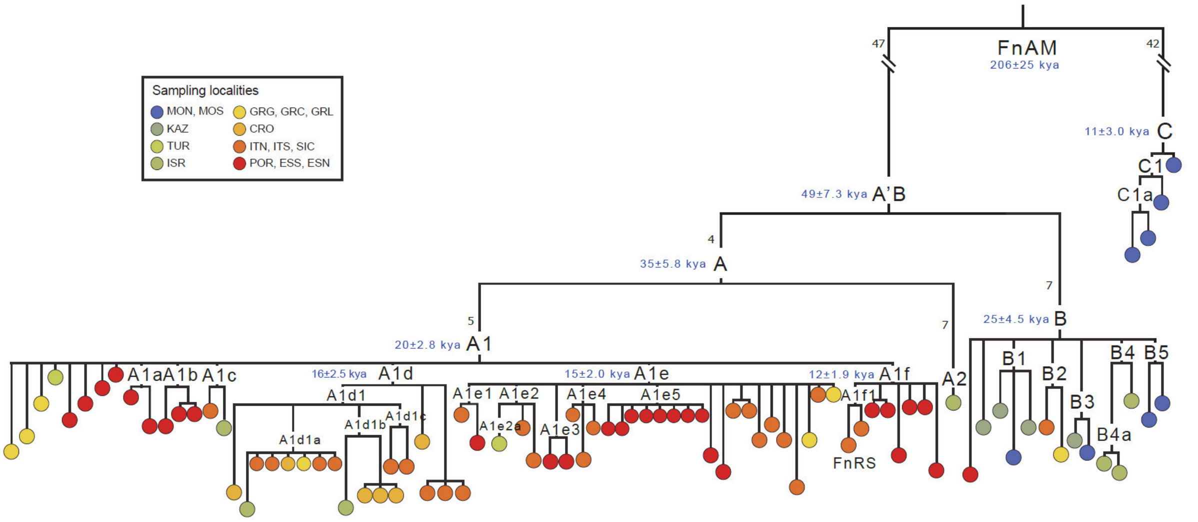

Supplementary Fig. 17 | Schematic maximum parsimony phylogeny of 89 lesser kestrel mitogenomes. The tree was rooted using the common kestrel (*Falco tinnunculus*) reference mitogenome. Numbers of nucleotide substitutions for the deepest branches are shown as well as haplogroup and sub-haplogroup affiliations (above nodes). Except for A2, sub-haplogroups were named only when encompassing at least two haplotypes. Coalescent age estimates for major nodes are reported. FnRS indicates the *F. naumanni* reference sequence. FnAM indicates the reconstructed *F. naumanni* ancestral mitogenome. Abbreviations for sampling localities are defined in Supplementary Table 1.

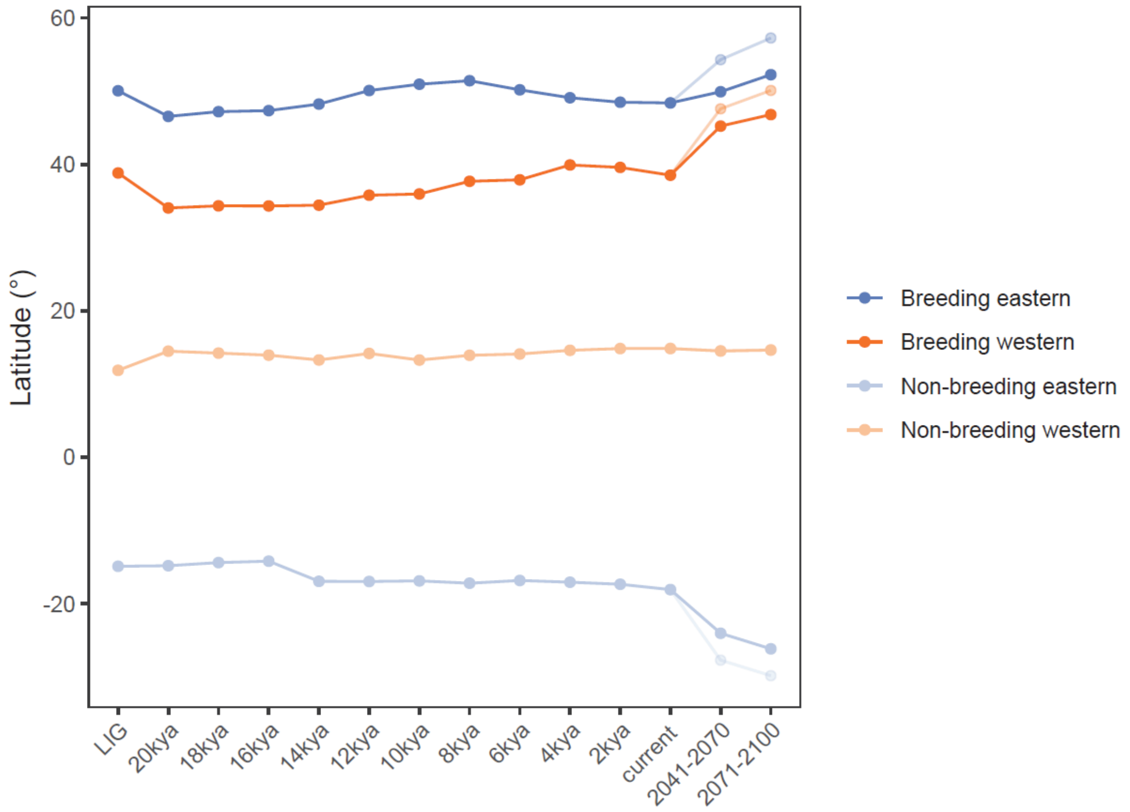

Supplementary Fig. 18 | Temporal trends for the latitude of the centroid of the distribution range for both Western and Eastern evolutionarily significant units (ESUs) during the breeding and non-breeding seasons. Future values are based on projections under ‘extreme warming’ (UKESM1-0-LL; SSP5-8.5) (faded circles/lines) or ‘moderate warming’ future climate (GFDL-ESM4; SSP3-7.0) (dark circles/lines). LIG: last interglacial period (approx. 120,000-140,000 years BP). Data are provided at https://doi.org/10.5281/zenodo.14988067.

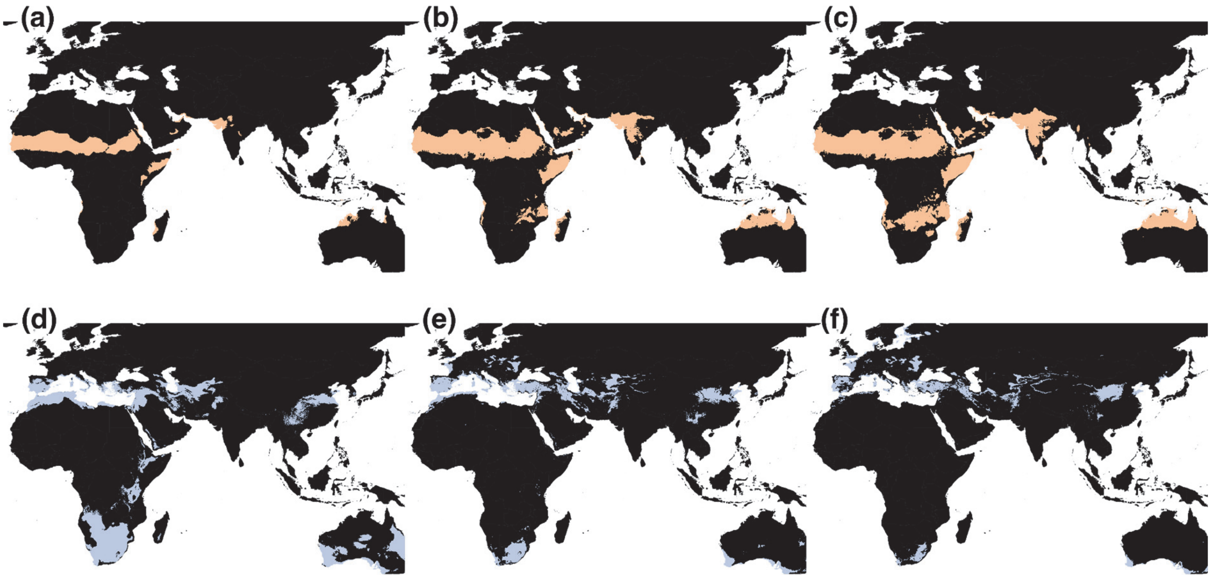

Supplementary Fig. 19 | Predicted non-breeding range for the Western and Eastern lesser kestrel evolutionarily significant units (ESUs). Predicted ranges in the present **(a, d)** and in the future (2041-2070 **[b, e]** and 2071-2100 **[c, f]**) using an 'extreme warming' future climate (UKESM1-0-LL; SSP5-8.5). This figure shows total predicted ranges before applying the 1,500 km-buffer around known occurrence locations (see Methods). Data are provided at https://doi.org/10.5281/zenodo.14988067. Background maps were obtained from the rnaturalearth v.0.3.2 R package.

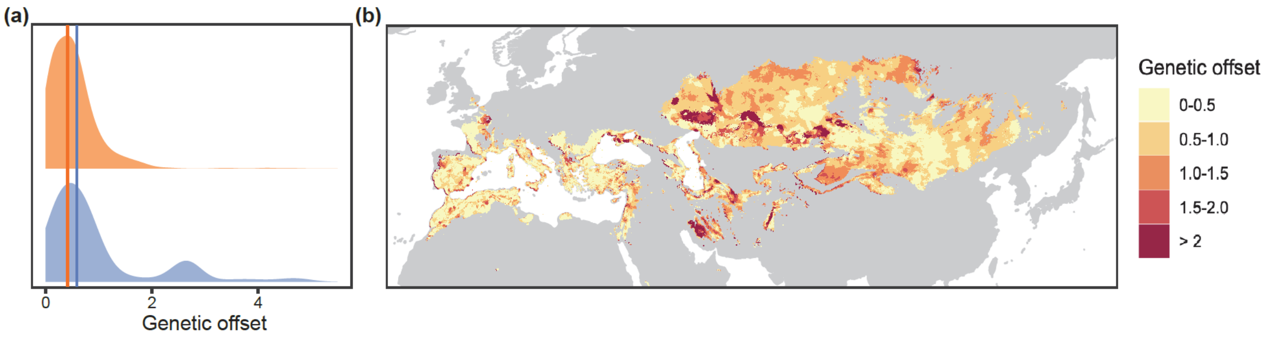

Supplementary Fig. 20 | Genetic offsets for lesser kestrel evolutionary lineages assuming 'moderate warming' future climate. a Density plots of predicted genetic offsets in 2041-2070 for all breeding localities within the Western and Eastern evolutionarily significant units (ESUs) showing slightly higher offsets for the Eastern one. Median values for each ESU are shown as coloured vertical lines. **b** Predicted genetic offsets across the species current distribution range between the present and the 2041-2070 period based on projections assuming 'moderate warming' future climate (GFDL-ESM4; SSP3-7.0). The highest offsets are in the western and northern part of the Eastern ESU's range. Data are provided at https://doi.org/10.5281/zenodo.14988067. Background maps were obtained from the rnaturalearth v.0.3.2 R package.

Supplementary Tables

Supplementary Table 1. Details of lesser kestrel genetic samples for each sampling locality. Information includes locality acronym and its description, the evolutionarily significant unit (ESU) individuals of each locality were assigned to, geographic coordinates for each locality (average of sampling sites for that locality; see legend to Supplementary Data 1) and the number of individuals available for genomic (double-digest Restriction-Site Associated DNA [ddRAD]) or mitogenomic (mtDNA) analyses. Localities are sorted by increasing longitude (west to east).

Acronym	Description	ESU	Longitude	Latitude	ddRAD	mtDNA
POR	Portugal	Western	-7.93	37.69	-	14
ESS	Southern Spain	Western	-6.49	37.40	7	8
ESN	Northern Spain	Western	-6.40	39.52	8	7
ITN	Northern Italy	Western	11.37	44.80	7	11
SIC	Sicily	Western	14.14	37.49	6	1
CRO	Croatia	Western	14.79	44.76	3	5
ITS	Southern Italy	Western	16.60	40.67	8	14
GRG	Western Greece	Western	20.85	39.67	3	2
GRC	Central Greece	Western	22.61	39.52	5	4
GRL	Eastern Greece	Western	25.29	39.91	6	2
TUR	Turkey	Western	34.04	39.33	3	2
ISR	Israel	Western	35.07	32.60	8	7
KAZ	Kazakhstan	Eastern	47.34	47.98	4	4
RUS	Southern Russia	Eastern	94.58	50.47	6	3
MOS	Southern Mongolia	Eastern	106.93	42.57	4	2
MON	Northern Mongolia	Eastern	110.51	48.16	6	6

Supplementary Table 2. Assembly statistics, BUSCO results and annotation statistics for the lesser kestrel reference genome.

**Genome assembly statistics**	
Genome size (Gbp)	1.22
Number of scaffolds	290
Number of chromosomes	25 autosomes + ZW + MT
Genomic sequence assigned to chromosomes	98.92%
Scaffold N50 (Mbp)	91.8
Scaffold L50	6
Maximum scaffold length (Mbp)	127
GC percent	42
BUSCO (vertebrata_odb10)	C: 96.8% [S: 95.7%,D: 1.1%], F: 1.2%, M: 2.0%
Genes and pseudogenes	19,775
Protein-coding genes	16,079
Repeat content (Mbp)	263

Supplementary Table 3. Pooled land cover categories used in this study, the original land cover categories and their descriptions.

Pooled land cover category	Original land cover category	Description
Forest	Closed forest, evergreen needle leaf	Tree canopy >70 %, almost all needle leaf trees remain green all year. Canopy is never without green foliage
	Closed forest, deciduous needle leaf	Tree canopy >70 %, consists of seasonal needle leaf tree communities with an annual cycle of leaf-on and leaf-off periods
	Closed forest, evergreen, broad leaf	Tree canopy >70 %, almost all broadleaf trees remain green year round. Canopy is never without green foliage
	Closed forest, deciduous broad leaf	Tree canopy >70 %, consists of seasonal broadleaf tree communities with an annual cycle of leaf-on and leaf-off periods
	Closed forest, mixed	Closed forest, mix of types
	Closed forest, unknown	Closed forest, not matching any of the other definitions
	Open forest, evergreen needle leaf	Top layer- trees 15-70 % and second layer mixed of shrubs and grassland, almost all needle leaf trees remain green all year. Canopy is never without green foliage
	Open forest, deciduous needle leaf	Top layer- trees 15-70 % and second layer mixed of shrubs and grassland, consists of seasonal needle leaf tree communities with an annual cycle of leaf-on and leaf-off periods
	Open forest, evergreen broad leaf	Top layer- trees 15-70 % and second layer mixed of shrubs and grassland, almost all broadleaf trees remain green year round. Canopy is never without green foliage
	Open forest, deciduous broad leaf	Top layer- trees 15-70 % and second layer mixed of shrubs and grassland, consists of seasonal broadleaf tree communities with an annual cycle of leaf-on and leaf-off periods
	Open forest, mixed	Open forest, mix of types
	Open forest, unknown	Open forest, not matching any of the other definitions
Shrubs	Shrubs	Woody perennial plants with persistent and woody stems and without any defined main stem being less than 5 m tall. The shrub foliage can be either evergreen or deciduous
Herbaceous vegetation	Herbaceous vegetation	Plants without persistent stem or shoots above ground and lacking definite firm structure. Tree and shrub cover is less than 10 %
	Herbaceous wetland	Lands with a permanent mixture of water and herbaceous or woody vegetation. The vegetation can be present in either salt, brackish, or fresh water
Bare	Bare / sparse vegetation	Lands with exposed soil, sand, or rocks and never has more than 10 % vegetated cover during any time of the year
Cropland	Cultivated and managed vegetation/agriculture (cropland)	Lands covered with temporary crops followed by harvest and a bare soil period (e.g., single and multiple cropping systems). Note that perennial woody crops will be classified as the appropriate forest or shrub land cover type
Urban	Urban / built up	Land covered by buildings and other manmade structures
Other	Moss and lichen	Moss and lichens
	Snow and Ice	Lands under snow or ice cover throughout the year
	Permanent water bodies	Lakes, reservoirs, and rivers. Can be either fresh- or saltwater
	Open sea	Oceans, seas. Can be either fresh- or saltwater

Supplementary Table 4. List of genes with climate-associated single-nucleotide polymorphisms (SNPs) in the lesser kestrel. These genes are potentially associated with processes that could be involved in divergent selection between lesser kestrel Western and Eastern evolutionarily significant units (ESUs).

Gene	Processes	References	F_{ST}	Chrom.	Position
AATF	thermal adaptation	Paim *et al.* [61]	0.09	1	110,958,111
ADORA2A	adaptation to high altitude	Granka *et al.* [62]	0.46	1	88,972,659
ALS2	memory formation	Benjelloun *et al.* [63]	0.19	8	52,029,502
ANKS6	adaptation to high altitude	Beckman *et al.* [64]	0.12	3	48,998,265
CACNA1C	adaptation to high altitude; circadian rhythm	Zhang *et al.* [65], Nudell *et al.* [66]	0.18	5	23,819,707
CASK	adaptation to high altitude; memory formation	Wallberg *et al.* [67], Gillespie & Hodge [68]	0.06	2	83,204,973
CDH8	thermal adaptation	Ferris *et al.* [69]	0.22	15	8,559,930
CPNE4	thermal adaptation; differences in migratory routes	Günther & Coop [70], Delmore *et al.* [71]	0.12	4	1,711,128
DPP4	adaptation to high altitude	Wei *et al.* [72]	0.33;0.28	8	41,192,565; 41,197,033
EMP2	circadian rhythm	Forni *et al.* [73]	0.18	4	70,411,550
EPB41L2	adaptation to arid environments	Mwacharo *et al.* [74]	0.12	6	55,440,652
EVI5	adaptation to high altitude	Li *et al.* [75]	0.27	11	16,538,701
FAR1	lipid metabolism	Almuqbil *et al.* [76]	0.10;0.13	10	10,525,363; 10,527,201
FASN	thermal adaptation; lipid metabolism	Chen *et al.* [77], Jayakumar *et al.* [78]	0.40;0.31	1	127,269,272; 127,298,912
HERC1	thermal adaptation; differences in migratory routes	Paim *et al.* [61], Asaduzzaman *et al.* [79]	0.19	7	22,766,867
KCNMB2	thermal adaptation; differences in migratory routes	Delmore *et al.* [71], Weldenegodguad *et al.* [80]	0.12	13	21,763,163
LDB3	lipid metabolism	Garske *et al.* [81]	0.12	9	40,414,188
NEO1	memory formation	Branch *et al.* [82]	0.25	7	1,514,106
PAK3	differences in migratory routes	Cavedon *et al.* [83]	0.22	14	4,147,928
PTPN5	adaptation to high altitude	Qu *et al.* [84]	0.21	10	232,560
RNF43	adaptation to high altitude; lipid metabolism; differences in migratory routes	Micheletti *et al.* [85], Deng *et al.* [86], Belenguer *et al.* [87]	0.06	1	100,606,921
RORA	adaptation to high altitude; circadian rhythm	Foll *et al.* [88], Dall'Ara *et al.* [89]	0.11	7	21,564,660
SLC2A9	adaptation to arid environments	Marra *et al.* [90]	0.13	1	52,489,101
TACC3	thermal adaptation	Bentley *et al.* [91]	0.10	1	46,247,713
TECRL	adaptation to arid environments	Bhardwaj *et al.* [92]	0.10	1	65,498,010
ZBTB20	thermal adaptation; lipid metabolism; memory formation	Han *et al.* [93], Liu *et al.* [94], Nielsen *et al.* [95]	0.12	5	6,320,742

Supplementary Table 5. Codes and description of bioclimatic variables used in this study. Codes and descriptions as reported in the CHELSA v2.1 database [96].

Code	Description
BIO1	Annual mean temperature
BIO2	Mean diurnal range [mean of monthly (max temp - min temp)]
BIO3	Isothermality (BIO2/BIO7) (×100)
BIO4	Temperature seasonality (standard deviation ×100)
BIO5	Max temperature of warmest month
BIO6	Min temperature of coldest month
BIO7	Temperature annual range (BIO5-BIO6)
BIO8	Mean temperature of wettest quarter
BIO9	Mean temperature of driest quarter
BIO10	Mean temperature of warmest quarter
BIO11	Mean temperature of coldest quarter
BIO12	Annual precipitation
BIO13	Precipitation of wettest month
BIO14	Precipitation of driest month
BIO15	Precipitation seasonality (coefficient of variation)
BIO16	Precipitation of wettest quarter
BIO17	Precipitation of driest quarter
BIO18	Precipitation of warmest quarter
BIO19	Precipitation of coldest quarter

Supplementary Table 6. Oligonucleotides employed for long-range PCR amplification of lesser kestrel mitogenomes. The three primer pairs used to amplify fragments A, B and C are shown.

Fragment	Primer name	Sequence 5'-> 3'	Tm (°C)	GC %
A	Fn_16234 FOR	TAGATTCCAGCCGCAACTTC	60	50
	Fn_4910 REV	TTGGTGGGAGTGTGATTGTG	60	50
B	Fn_4730 FOR	CTCCATAGTAACCGCCTCCA	60	50
	Fn_10564 REV	AGGTTGGGATTAGGGTTGCT	60	50
C	Fn_10369 FOR	AACCACCTCCAACATGAACC	60	50
	Fn_16460 REV	TGCTGGTTGTTTTGGATACG	59	45

Supplementary Table 7. Summary of breeding and non-breeding occurrence records of lesser kestrels. Datasets are listed in decreasing order of numerical importance. No country/region is reported if a given dataset encompassed more than a single country/region.

Dataset	Country/region	N records	Reference
**Western ESU**			
Breeding records			
SEO census	Spain	547	Bustamante [97]
LIFE FALKON archive	Italy	415	Morganti *et al.* [98]; M. Morganti, unpubl. data
eBird breeding	-	326	eBird [36]
GBIF breeding	-	185	GBIF.org [35]
Genetic samples collection localities	-	97	this study (Supplementary Table 1)
Breeding sites of tracked birds	-	94	Sarà *et al.* [37]
Portugal	Portugal	70	I. Catry, unpubl. data
Greece	Greece	68	T. Bounas, unpubl. data
RU-BIRDS.RU	-	3	Voltzit [99]
Armenia	Armenia	1	Ananian [100]
Non-breeding records			
GBIF non-breeding	Sahel	118	GBIF.org [38]
Non-breeding areas of tracked birds	Sahel	111	Sarà *et al.* [37]
**Eastern ESU**			
Breeding records			
GBIF breeding	-	309	GBIF.org [35]
Kazakhstan	Kazakhstan	119	E. Bragin, A. Bragin and T. Katzner, unpubl. data
Mongolia	Mongolia	99	B. Daavasuren and N. Batbayar, unpubl. data
RU-BIRDS.RU	-	88	Voltzit [99]
Genetic samples collection localities	-	23	this study (Supplementary Table 1)
eBird breeding	-	19	eBird [36]
Non-breeding records			
GBIF non-breeding	southern Africa	3933	GBIF.org [38]
Non-breeding areas of tracked birds	southern Africa	3	Milhouser [101]

Supplementary Table 8. Permutation importance of selected bioclimatic variables included in lesser kestrel species distribution models (SDMs). Values obtained from MaxEnt models based on 10 permutations, calculated using the command provided in the R package SDMTune [102] built for each season (breeding, non-breeding) and lineage (evolutionarily significant unit, ESU).

Season/ESU	Variable	Permutation importance (s.d.)
Breeding West	BIO1	67.2 (0.009)
	BIO7	15.6 (0.003)
	BIO12	16.6 (0.009)
	BIO15	0.60 (0.001)
Breeding East	BIO1	69.2 (0.020)
	BIO7	14.2 (0.004)
	BIO12	15.9 (0.004)
	BIO15	0.80 (0.001)
Non-breeding West	BIO1	11.9 (0.004)
	BIO7	10.3 (0.006)
	BIO12	5.2 (0.006)
	BIO15	72.6 (0.020)
Non-breeding East	BIO1	59.7 (0.009)
	BIO7	9.2 (0.003)
	BIO12	20.3 (0.005)
	BIO15	10.8 (0.003)

Supplementary Table 9. Priors for DIYABC demographic modelling of lesser kestrel populations. All priors were set with a uniform distribution. We defined the following temporal constraints: t3 > t2 > t1.

Parameter	Parameter description	Prior parameters	
		Minimum	Maximum
NC	Ancestral population size	10	1,000,000
N4	Population size of Asian	10	1,000,000
N3	Population size of Middle East	10	1,000,000
N2	Population size of Central and Eastern Europe	10	1,000,000
N1	Population size of Iberia	10	1,000,000
t1	Time of admixture in Central and Eastern Europe	0	10,000
t2	Time of admixture in Middle East	0	50,000
t3	Time of split between Western and Eastern ESUs	10	100,000
r1	Admixture rate from Iberia to Central and Eastern Europe	0.05	0.95
r2	Admixture rate from Europe to Middle East	0.05	0.95

Supplementary References

1. Rhie, A. *et al.* Towards complete and error-free genome assemblies of all vertebrate species. *Nature* **592**, 737–746 (2021).

2. Koren, S. *et al.* De novo assembly of haplotype-resolved genomes with trio binning. *Nat. Biotechnol.* **36**, 1174–1182 (2018).

3. Ranallo-Benavidez, T. R., Jaron, K. S. & Schatz, M. C. GenomeScope 2.0 and Smudgeplot for reference-free profiling of polyploid genomes. *Nat. Commun.* **11**, 1432 (2020).

4. Rhie, A., Walenz, B. P., Koren, S. & Phillippy, A. M. Merqury: reference-free quality, completeness, and phasing assessment for genome assemblies. *Genome Biol.* **21**, 245 (2020).

5. Guan, D. *et al.* Identifying and removing haplotypic duplication in primary genome assemblies. *Bioinformatics* **36**, 2896–2898 (2020).

6. Ghurye, J. *et al.* Integrating Hi-C links with assembly graphs for chromosome-scale assembly. *PLoS Comput. Biol.* **15**, e1007273 (2019).

7. Chow, W. *et al.* gEVAL - a web-based browser for evaluating genome assemblies. *Bioinformatics* **32**, 2508–2510 (2016).

8. Howe, K., Chow, W., Collins, J. & Others. Significantly improving the quality of genome assemblies through curation. *GigaScience* **10**, giaa153 (2021).

9. Pruitt, K. D. *et al.* RefSeq: an update on mammalian reference sequences. *Nucleic Acids Res.* **42**, D756–63 (2014).

10. Morgulis, A., Gertz, E. M., Schäffer, A. A. & Agarwala, R. WindowMasker: window-based masker for sequenced genomes. *Bioinformatics* **22**, 134–141 (2006).

11. Smit, A. F. A., Hubley, R & Green, P. *RepeatMasker Open-4.0*. http://www.repeatmasker.org (2015).

12. Storer, J., Hubley, R., Rosen, J., Wheeler, T. J. & Smit, A. F. The Dfam community resource of transposable element families, sequence models, and genome annotations. *Mob. DNA* **12**, 2 (2021).

13. Bao, W., Kojima, K. K. & Kohany, O. Repbase Update, a database of repetitive elements in eukaryotic genomes. *Mob. DNA* **6**, 11 (2015).

14. Simão, F. A., Waterhouse, R. M., Ioannidis, P., Kriventseva, E. V. & Zdobnov, E. M. BUSCO: assessing genome assembly and annotation completeness with single-copy orthologs. *Bioinformatics* **31**, 3210–3212 (2015).

15. Li, H. *et al.* The Sequence Alignment/Map format and SAMtools. *Bioinformatics* **25**, 2078–2079 (2009).

16. Li, H. A statistical framework for SNP calling, mutation discovery, association mapping and population genetical parameter estimation from sequencing data. *Bioinformatics* **27**, 2987–2993 (2011).

17. Danecek, P. *et al.* Twelve years of SAMtools and BCFtools. *Gigascience* **10**, (2021).

18. Rochette, N. C., Rivera-Colón, A. G. & Catchen, J. M. Stacks 2: Analytical methods for paired-end sequencing improve RADseq-based population genomics. *Mol. Ecol.* **28**, 4737–4754 (2019).

19. Maruki, T. & Lynch, M. Genotype-frequency estimation from high-throughput sequencing data. *Genetics* **201**, 473–486 (2015).

20. Maruki, T. & Lynch, M. Genotype calling from population-genomic sequencing data. *G3: Genes|Genomes|Genetics* **7**, 1393–1404 (2017).

21. Danecek, P. *et al.* The variant call format and VCFtools. *Bioinformatics* **27**, 2156–2158 (2011).

22. Linck, E. & Battey, C. J. Minor allele frequency thresholds strongly affect population structure inference with genomic data sets. *Mol. Ecol. Resour.* **19**, 639–647 (2019).

23. Untergasser, A. *et al.* Primer3—new capabilities and interfaces. *Nucleic Acids Res.* **40**, e115–e115 (2012).

24. Formenti, G. *et al.* Complete vertebrate mitogenomes reveal widespread repeats and gene duplications. *Genome Biol.* **22**, 120 (2021).

25. Petkova, D., Novembre, J. & Stephens, M. Visualizing spatial population structure with estimated effective migration surfaces. *Nat. Genet.* **48**, 94–100 (2016).

26. Malinsky, M., Trucchi, E., Lawson, D. J. & Falush, D. RADpainter and fineRADstructure: Population Inference from RADseq Data. *Mol. Biol. Evol.* **35**, 1284–1290 (2018).

27. Wilson, G. A. & Rannala, B. Bayesian inference of recent migration rates using multilocus genotypes. *Genetics* **163**, 1177–1191 (2003).

28. Mussmann, S. M., Douglas, M. R., Chafin, T. K. & Douglas, M. E. BA3-SNPs: Contemporary migration reconfigured in BayesAss for next-generation sequence data. *Methods Ecol. Evol.* **10**, 1808–1813 (2019).

29. Rambaut, A., Drummond, A. J., Xie, D., Baele, G. & Suchard, M. A. Posterior Summarization in Bayesian Phylogenetics Using Tracer 1.7. *Syst. Biol.* **67**, 901–904 (2018).

30. Bryant, D. & Moulton, V. Neighbor-net: an agglomerative method for the construction of phylogenetic networks. *Mol. Biol. Evol.* **21**, 255–265 (2004).

31. Huson, D. H. & Bryant, D. Application of phylogenetic networks in evolutionary studies. *Mol. Biol. Evol.* **23**, 254–267 (2006).

32. Knaus, B. J. & Grünwald, N. J. vcfr: a package to manipulate and visualize variant call format data in R. *Mol. Ecol. Resour.* **17**, 44–53 (2017).

33. Paradis, E. & Schliep, K. ape 5.0: an environment for modern phylogenetics and evolutionary analyses in R. *Bioinformatics* **35**, 526–528 (2019).

34. Pockrandt, C., Alzamel, M., Iliopoulos, C. S. & Reinert, K. GenMap: ultra-fast computation of genome mappability. *Bioinformatics* **36**, 3687–3692 (2020).

35. GBIF.org *GBIF Occurrence Download.* https://doi.org/10.15468/dl.jhfvad (2021).

36. eBird *eBird: An online database of bird distribution and abundance [web application]*. http://www.ebird.org (2021).

37. Sarà, M. *et al.* Broad-front migration leads to strong migratory connectivity in the lesser kestrel (*Falco naumanni*). *J. Biogeogr.* **46**, 2663–2677 (2019).

38. GBIF.org. *GBIF Occurrence Download.* https://doi.org/10.15468/dl.fwrtg (2021).

39. Muscarella, R. *et al.* ENMeval: An R package for conducting spatially independent evaluations and estimating optimal model complexity forMaxentecological niche models. *Methods Ecol. Evol.* **5**, 1198–1205 (2014).

40. Elith, J. *et al.* A statistical explanation of MaxEnt for ecologists. *Divers. Distrib.* **17**, 43–57 (2011).

41. Grimmett, L., Whitsed, R. & Horta, A. Presence-only species distribution models are sensitive to sample prevalence: Evaluating models using spatial prediction stability and accuracy metrics. *Ecol. Modell.* **431**, 109194 (2020).

42. Merow, C. *et al.* What do we gain from simplicity versus complexity in species distribution models? *Ecography* **37**, 1267–1281 (2014).

43. Brambilla, M. *et al.* Identifying climate refugia for high-elevation Alpine birds under current climate warming predictions. *Glob. Chang. Biol.* **28**, 4276–4291 (2022).

44. Valavi, R., Elith, J., Lahoz-Monfort, J. J. & Guillera-Arroita, G. Flexible species distribution modelling methods perform well on spatially separated testing data. *Glob. Ecol. Biogeogr.* **32**, 369–383 (2023).

45. Warren, D. L. & Seifert, S. N. Ecological niche modeling in Maxent: the importance of model complexity and the performance of model selection criteria. *Ecol. Appl.* **21**, 335–342 (2011).

46. Liu, C., White, M. & Newell, G. Selecting thresholds for the prediction of species occurrence with presence-only data. *J. Biogeogr.* **40**, 778–789 (2013).

47. Lasky, J. R. *et al.* Characterizing genomic variation of *Arabidopsis thaliana*: the roles of geography and climate: geography, climate and arabidopsis genomics. *Mol. Ecol.* **21**, 5512–5529 (2012).

48. Gautier, M. Genome-wide scan for adaptive divergence and association with population-specific covariates. *Genetics* **201**, 1555–1579 (2015).

49. Caye, K., Jumentier, B., Lepeule, J. & François, O. LFMM 2: Fast and Accurate Inference of Gene-Environment Associations in Genome-Wide Studies. *Mol. Biol. Evol.* **36**, 852–860 (2019).

50. Lotterhos, K. E. The effect of neutral recombination variation on genome scans for selection. *G3* **9**, 1851–1867 (2019).

51. Booker, T. R. The structure of the environment influences the patterns and genetics of local adaptation. *Evol. Lett.* **8**, 787–798 (2024).

52. Whitlock, M. C. & Lotterhos, K. E. Reliable Detection of Loci Responsible for Local Adaptation: Inference of a Null Model through Trimming the Distribution of F(ST). *Am. Nat.* **186 Suppl 1**, S24–36 (2015).

53. Luu, K., Bazin, E. & Blum, M. G. B. pcadapt: an R package to perform genome scans for selection based on principal component analysis. *Mol. Ecol. Resour.* **17**, 67–77 (2017).

54. Storey, M. J. D., Bass, A. J., Dabney, A. & Robinson D. *qvalue: Q-value estimation for false discovery rate control. R package version 2.38.0*. http://github.com/jdstorey/qvalue (2024).

55. Lotterhos, K. E. & Whitlock, M. C. The relative power of genome scans to detect local adaptation depends on sampling design and statistical method. *Mol. Ecol.* **24**, 1031–1046 (2015).

56. Bouckaert, R. *et al.* BEAST 2.5: An advanced software platform for Bayesian evolutionary analysis. *PLoS Comput. Biol.* **15**, e1006650 (2019).

57. Xu, B. & Yang, Z. PAMLX: a graphical user interface for PAML. *Mol. Biol. Evol.* **30**, 2723–2724 (2013).

58. Olivieri, A. *et al.* Mitogenomes from Egyptian cattle breeds: New clues on the origin of haplogroup Q and the early spread of Bos taurus from the Near East. *PLoS One* **10**, e0141170 (2015).

59. Lombardo, G. *et al.* The mitogenome relationships and phylogeography of barn swallows (*Hirundo rustica*). *Mol. Biol. Evol.* **39**, msac113 (2022).

60. Fuchs, J., Johnson, J. A. & Mindell, D. P. Rapid diversification of falcons (Aves: Falconidae) due to expansion of open habitats in the Late Miocene. *Mol. Phylogenet. Evol.* **82 Pt A**, 166–182 (2015).

61. Paim, T. do P., Alves dos Santos, C., Faria, D. A. de, Paiva, S. R. & McManus, C. Genomic selection signatures in Brazilian sheep breeds reared in a tropical environment. *Livest. Sci.* **258**, 104865 (2022).

62. Granka, J. M. *et al.* Limited evidence for classic selective sweeps in African populations. *Genetics* **192**, 1049–1064 (2012).

63. Benjelloun, B. *et al.* Multiple adaptive solutions to face climatic constraints: novel insights in the debate over the role of convergence in local adaptation. *bioRxiv* 2021.11.18.469099 (2021) doi:10.1101/2021.11.18.469099.

64. Beckman, E. J. *et al.* The genomic basis of high-elevation adaptation in wild house mice (*Mus musculus domesticus*) from South America. *Genetics* **220**, (2022).

65. Zhang, T. *et al.* Phenotypic and genomic adaptations to the extremely high elevation in plateau zokor (*Myospalax baileyi*). *Mol. Ecol.* **30**, 5765–5779 (2021).

66. Nudell, V. *et al.* Entrainment of circadian rhythms to temperature reveals amplitude deficits in fibroblasts from patients with bipolar disorder and possible links to calcium channels. *Mol Neuropsychiatry* **5**, 115–124 (2019).

67. Wallberg, A., Schöning, C., Webster, M. T. & Hasselmann, M. Two extended haplotype blocks are associated with adaptation to high altitude habitats in East African honey bees. *PLoS Genet.* **13**, e1006792 (2017).

68. Gillespie, J. M. & Hodge, J. J. L. CASK regulates CaMKII autophosphorylation in neuronal growth, calcium signaling, and learning. *Front. Mol. Neurosci.* **6**, 27 (2013).

69. Ferris, K. G. *et al.* The genomics of rapid climatic adaptation and parallel evolution in North American house mice. *PLoS Genet.* **17**, e1009495 (2021).

70. Günther, T. & Coop, G. Robust identification of local adaptation from allele frequencies. *Genetics* **195**, 205–220 (2013).

71. Delmore, K. E. *et al.* Genomic analysis of a migratory divide reveals candidate genes for migration and implicates selective sweeps in generating islands of differentiation. *Molec. Ecol.* **24**, 1873–1888 (2015).

72. Wei, C. *et al.* Genome-wide analysis reveals adaptation to high altitudes in Tibetan sheep. *Sci. Rep.* **6**, 26770 (2016).

73. Forni, D. *et al.* Genetic adaptation of the human circadian clock to day-length latitudinal variations and relevance for affective disorders. *Genome Biol.* **15**, 499 (2014).

74. Mwacharo, J. M. *et al.* Genomic footprints of dryland stress adaptation in Egyptian fat-tail sheep and their divergence from East African and western Asia cohorts. *Sci. Rep.* **7**, 17647 (2017).

75. Li, D. *et al.* Population genomics identifies patterns of genetic diversity and selection in chicken. *BMC Genomics* **20**, 263 (2019).

76. Almuqbil, M., AbuMelha, A. & Albokhari, D. Milder presentation of autosomal dominant fatty acyl CoA reductase 1-related syndrome: Report of the first Middle Eastern patient and review of the literature. *Clin Case Rep* **10**, e6307 (2022).

77. Chen, Y. *et al.* The combination of genomic offset and niche modelling provides insights into climate change-driven vulnerability. *Nat. Commun.* **13**, 4821 (2022).

78. Jayakumar, A. *et al.* Human fatty acid synthase: properties and molecular cloning. *Proc. Natl. Acad. Sci. U. S. A.* **92**, 8695–8699 (1995).

79. Asaduzzaman, M. *et al.* Morpho-Genetic Divergence and Adaptation of Anadromous Hilsa shad (*Tenualosa ilisha*) Along Their Heterogenic Migratory Habitats. *Frontiers in Marine Science* **7**, (2020).

80. Weldenegodguad, M. *et al.* Genome sequence and comparative analysis of reindeer (*Rangifer tarandus*) in northern Eurasia. *Sci. Rep.* **10**, 8980 (2020).

81. Garske, K. M. *et al.* Reverse gene-environment interaction approach to identify variants influencing body-mass index in humans. *Nat Metab* **1**, 630–642 (2019).

82. Branch, C. L. *et al.* The genetic basis of spatial cognitive variation in a food-caching bird. *Curr. Biol.* **32**, 210–219 (2021).

83. Cavedon, M. *et al.* Genomic legacy of migration in endangered caribou. *PLoS Genet.* **18**, e1009974 (2022).

84. Qu, Y. *et al.* Genetic responses to seasonal variation in altitudinal stress: whole-genome resequencing of great tit in eastern Himalayas. *Sci. Rep.* **5**, 14256 (2015).

85. Micheletti, S. J., Matala, A. R., Matala, A. P. & Narum, S. R. Landscape features along migratory routes influence adaptive genomic variation in anadromous steelhead (*Oncorhynchus mykiss*). *Mol. Ecol.* **27**, 128–145 (2018).

86. Deng, L. *et al.* Prioritizing natural-selection signals from the deep-sequencing genomic data suggests multi-variant adaptation in Tibetan highlanders. *Natl Sci Rev* **6**, 1201–1222 (2019).

87. Belenguer, G. *et al.* RNF43/ZNRF3 loss predisposes to hepatocellular-carcinoma by impairing liver regeneration and altering the liver lipid metabolic ground-state. *Nat. Commun.* **13**, 334 (2022).

88. Foll, M., Gaggiotti, O. E., Daub, J. T., Vatsiou, A. & Excoffier, L. Widespread signals of convergent adaptation to high altitude in Asia and america. *Am. J. Hum. Genet.* **95**, 394–407 (2014).

89. Dall'Ara, I. *et al.* Demographic history and adaptation account for clock gene diversity in humans. *Heredity* **117**, 165–172 (2016).

90. Marra, N. J., Romero, A. & DeWoody, J. A. Natural selection and the genetic basis of osmoregulation in heteromyid rodents as revealed by RNA-seq. *Mol. Ecol.* **23**, 2699–2711 (2014).

91. Bentley, B. P., Haas, B. J., Tedeschi, J. N. & Berry, O. Loggerhead sea turtle embryos (*Caretta caretta*) regulate expression of stress response and developmental genes when exposed to a biologically realistic heat stress. *Mol. Ecol.* **26**, 2978–2992 (2017).

92. Bhardwaj, S. *et al.* Genome-wide diversity analysis for signatures of selection of *Bos indicus* adaptability under extreme agro-climatic conditions of temperate and tropical ecosystems. *Anim. Genet.* **20**, 200115 (2021).

93. Han, Z.-Q. *et al.* Whole-genome resequencing of Japanese whiting (*Sillago japonica*) provide insights into local adaptations. *Zool Res* **42**, 548–561 (2021).

94. Liu, G. *et al.* Regulation of hepatic lipogenesis by the zinc finger protein Zbtb20. *Nat. Commun.* **8**, 14824 (2017).

95. Nielsen, J. V., Thomassen, M., Møllgård, K., Noraberg, J. & Jensen, N. A. Zbtb20 defines a hippocampal neuronal identity through direct repression of genes that control projection neuron development in the isocortex. *Cereb. Cortex* **24**, 1216–1229 (2014).

96. Karger, D. N. *et al.* Climatologies at high resolution for the earth's land surface areas. *Sci. Data* **4**, 170122 (2017).

97. Bustamante, J. Cernícalo primilla (*Falco naumanni*). in *SEO/BirdLife: Atlas de las aves en invierno en España 2007-2010* 36–47 (Ministerio de Agricultura, Alimentación y Medio Ambiente-SEO/BirdLife. Madrid, 2012).

98. Morganti, M., Preatoni, D. & Sarà, M. Climate determinants of breeding and wintering ranges of lesser kestrels in Italy and predicted impacts of climate change. *J. Avian Biol.* **48**, 1595–1607 (2017).

99. Voltzit, O. *Birds Observations Database from Russia and Neighboring Regions - Zoological Museum of M.V. Lomonosov Moscow State University*. http://ru-birds.ru/en/ (2021).

100. Ananian, V. On the distribution and ecology of the Lesser Kestrel *Falco naumanni* in Armenia. *Sandgrouse* **31**, 44–54 (2009).

101. Milhouser, P. *Following the migration of lesser kestrels of Mongolia*. https://www.slideshare.net/GISITR/2016-conservation-track-geolocation-by-light-following-the-migration-of-lesser-kestrels-of-mongolia-by-paul-milhouser (2016).

102. Vignali, S., Barras, A. G., Arlettaz, R. & Braunisch, V. SDMtune: An R package to tune and evaluate species distribution models. *Ecol. Evol.* **10**, 11488–11506 (2020).
