# Supplementary material for: Evolutionarily distinct lineages of a migratory bird of prey show divergent responses to climate change
Source: Nat Commun. 2025 Apr 13;16:3503. doi: 10.1038/s41467-025-58617-5 (PMC11993763; doi:10.1038/s41467-025-58617-5)
Supplement: Supplementary file 2 — Description of Addtional Supplementary Files [file 41467_2025_58617_MOESM2_ESM.pdf]

Description of Additional Supplementary Files

Supplementary Data 1. List of genetic samples of lesser kestrels used in this study, including sample ID, sampling site and locality details and coordinates, European Nucleotide Archive (ENA) accession numbers, and mtDNA haplogroups. Samples were collected at 21 sampling sites; nearby sites (within 80 km) were pooled into 16 sampling localities for ease of reference. ddRAD = double-digest Restriction-Site Associated DNA; mtDNA = mitochondrial DNA; WGS = whole-genome sequencing. Population acronyms are defined in Supplementary Table 1.

Supplementary Data 2. Genes with climate-associated SNPs and their reported associations with local adaptation, phenotypic traits involved in local adaptation, domestication/urban adaptation and stress response based on a literature search.

Supplementary Data 3. Breeding and non-breeding occurrence records for lesser kestrels after pooling records within the same 2.5 arc-minute grid cell and duplicate records. These were the occurrence data used for implementing species distribution models (SDMs).
