# Supplementary material for: Evolutionarily distinct lineages of a migratory bird of prey show divergent responses to climate change
Source: Nat Commun. 2025 Apr 13;16:3503. doi: 10.1038/s41467-025-58617-5 (PMC11993763; doi:10.1038/s41467-025-58617-5)
Supplement: Supplementary file 6 — Reporting Summary [file 41467_2025_58617_MOESM6_ESM.pdf]

nature portfolio

Corresponding author(s): Joan Ferrer Obiol and Diego Rubolini

Last updated by author(s): Feb 23, 2025

Reporting Summary

Nature Portfolio wishes to improve the reproducibility of the work that we publish. This form provides structure for consistency and transparency in reporting. For further information on Nature Portfolio policies, see our Editorial Policies and the Editorial Policy Checklist.

Statistics

For all statistical analyses, confirm that the following items are present in the figure legend, table legend, main text, or Methods section.

n/a	Confirmed	
☐	☒	The exact sample size (*n*) for each experimental group/condition, given as a discrete number and unit of measurement
☐	☒	A statement on whether measurements were taken from distinct samples or whether the same sample was measured repeatedly
☐	☒	The statistical test(s) used AND whether they are one- or two-sided<br>*Only common tests should be described solely by name; describe more complex techniques in the Methods section.*
☒	☐	A description of all covariates tested
☒	☐	A description of any assumptions or corrections, such as tests of normality and adjustment for multiple comparisons
☐	☒	A full description of the statistical parameters including central tendency (e.g. means) or other basic estimates (e.g. regression coefficient) AND variation (e.g. standard deviation) or associated estimates of uncertainty (e.g. confidence intervals)
☐	☒	For null hypothesis testing, the test statistic (e.g. *F*, *t*, *r*) with confidence intervals, effect sizes, degrees of freedom and *P* value noted<br>*Give P values as exact values whenever suitable.*
☐	☒	For Bayesian analysis, information on the choice of priors and Markov chain Monte Carlo settings
☒	☐	For hierarchical and complex designs, identification of the appropriate level for tests and full reporting of outcomes
☐	☒	Estimates of effect sizes (e.g. Cohen's *d*, Pearson's *r*), indicating how they were calculated

Our web collection on statistics for biologists contains articles on many of the points above.

Software and code

Policy information about availability of computer code

Data collection	No software was used for data collection.
Data analysis	All code, scripts and additional data to reproduce the analyses conducted in this study are available on GitHub at the following link: https://doi.org/10.5281/zenodo.14988067<br><br>List of software used for this study:<br>GenomeScope v.2.0<br>Meryl v.1.3<br>TrioCanu v.2.1.1<br>Arrow (smrtanalysis 5.1.0.26412)<br>purge_dups v.1.2.6<br>scaff10x v2.0-2.1<br>Bionano Solve v3.2.1<br>Salsa 2.2<br>gEVAL<br>Eukaryotic Genome Annotation Pipeline v.8<br>Windowmasker v1.0.1<br>RepeatMasker 4.1.0<br>BUSCO 4.1.4<br>Stacks v2.0<br>BWA mem v.0.7.17

SAMtools v.1.10
VCFtools v.0.1.16
Primer3Plus v.3.3.0
bcl2fastq2 v.2.20
TrimGalore v.0.6.4
mitoVGP v.2.2
Geneious v8.0.5
FastQC v.0.11.5
Picard tools v2.18.29
BCFtools v1.15.1
PLINK v.1.9
Admixture v.1.3
fineRADstructure v.0.3
EEMS
BayesAss3-SNPs v.1.1
Treemix v.1.13
RADinitio v1.1.1
SplitsTree5 v.5.0.16
mtPhyl v.5.003
Fitchi v.1.1.4
Tracer v.1.7.1
R package vcfR v1.4.0
R package ape v5.7-1
GenMap v1.3.0
R package ENMeval v.2.0.4
R package phuassess v1.1
R package elevatr v0.4.2
R package ecospat v.4.0.0
R package vegan v.2.6-4
R package ecodist v.2.1.3
R package OutFLANK v.0.2
R package PCAdapt v.4.3.3
SnpEff v5.1
R package gplots v.3.1.3
R package qvalue v.2.16
R package gradientForest v0.1-32
DIYABC Random Forest v.1.0
Beast v.2.6.3
PAMLX v.1.3.1
MSMC2 v2.1.1
WhatsHap v1.4
SHAPEIT4 v4.1.2

For manuscripts utilizing custom algorithms or software that are central to the research but not yet described in published literature, software must be made available to editors and reviewers. We strongly encourage code deposition in a community repository (e.g. GitHub). See the Nature Portfolio guidelines for submitting code & software for further information.

Data

Policy information about availability of data

All manuscripts must include a data availability statement. This statement should provide the following information, where applicable:

- Accession codes, unique identifiers, or web links for publicly available datasets
- A description of any restrictions on data availability
- For clinical datasets or third party data, please ensure that the statement adheres to our policy

Data accession details are described in the manuscript under Data availability section. The lesser kestrel reference genome is available through NCBI (accession numbers: GCF_017639655.2 (primary) and GCA_017639645.1 (alternate)) along with NCBI Falco naumanni Annotation Release of the primary assembly (NCBI Falco naumanni Annotation Release 100). Population-level ddRAD, mitogenome and WGS data are archived on the European Nucleotide Archive (ENA) under accession number PRJEB71106. Details of genetic samples, results of literature searches for genes with SNPs associated with climate, and breeding and non-breeding occurrence records used for SDMs are provided as Supplementary Data files. Source data are provided with this paper at https://doi.org/10.5281/zenodo.14988067. The reference genome of Falco tinnunculus was donwloaded from the NCBI ftp site. CHELSA and Wordclim data were downloaded using wget, and land cover data were obtained from the Copernicus Global Land Service. Lesser kestrel occurrence data were mostly downloaded from the GBIF and eBird databases, with the addition of some other published/unpublished sources.

Research involving human participants, their data, or biological material

Policy information about studies with human participants or human data. See also policy information about sex, gender (identity/presentation), and sexual orientation and race, ethnicity and racism.

Reporting on sex and gender	Our study does not involve human subjects.
Reporting on race, ethnicity, or	*Please specify the socially constructed or socially relevant categorization variable(s) used in your manuscript and explain why they were used. Please note that such variables should not be used as proxies for other socially constructed/relevant variables*

other socially relevant groupings	*(for example, race or ethnicity should not be used as a proxy for socioeconomic status). Provide clear definitions of the relevant terms used, how they were provided (by the participants/respondents, the researchers, or third parties), and the method(s) used to classify people into the different categories (e.g. self-report, census or administrative data, social media data, etc.) Please provide details about how you controlled for confounding variables in your analyses.*
Population characteristics	*Describe the covariate-relevant population characteristics of the human research participants (e.g. age, genotypic information, past and current diagnosis and treatment categories). If you filled out the behavioural & social sciences study design questions and have nothing to add here, write "See above."*
Recruitment	*Describe how participants were recruited. Outline any potential self-selection bias or other biases that may be present and how these are likely to impact results.*
Ethics oversight	*Identify the organization(s) that approved the study protocol.*

Note that full information on the approval of the study protocol must also be provided in the manuscript.

Field-specific reporting

Please select the one below that is the best fit for your research. If you are not sure, read the appropriate sections before making your selection.

☐ Life sciences ☐ Behavioural & social sciences ☒ Ecological, evolutionary & environmental sciences

For a reference copy of the document with all sections, see nature.com/documents/nr-reporting-summary-flat.pdf

Ecological, evolutionary & environmental sciences study design

All studies must disclose on these points even when the disclosure is negative.

Study description	We provide an assessment of the vulnerability of the lesser kestrel to climate change by (1) inferring intraspecific evolutionary lineages and assessing their ecological differentiation, and (2) investigating lineage-specific genomic, demographic and distributional responses to past, present and future climatic fluctuations across its global distribution range.
Research sample	Genetic data were obtained from blood samples collected from 122 breeding adults (both males and females) and nestlings (sampled blindly regarding to sex) lesser kestrels. Sample details including sampling locality, coordinates, sampling year, sex, age and relatedness are reported in Supplementary Data 1. We did not consider sex in the genetic analyses because sampling was conducted irrespective of sex, both sexes were similarly represented in the sample (see 'Reporting on sex' section below), and we did not expect sex to affect population genetic structure at the global range scale. However, we removed genetic variants located on sex-determining chromosomes to ensure that population structure analyses were not biased by sex or by the sex composition of the sample.
Sampling strategy	We planned to obtain blood samples for genetic analyses from unrelated individuals across the entire breeding range of the species. In most cases, to avoid including strictly related individuals, we sampled a single nestling per brood, and avoided collecting samples from both parents and their offspring (with the exception of the samples used for generating the reference genome, which were two parents and one daughter). The spatial density of sampling localities was lower in Asia because some regions were challenging to sample due to limited accessibility. We implemented a high-resolution sequencing strategy to partly mitigate the low precision in statistical inference due to the low sample size. We also sequenced all samples at high sequencing depth (>30x) to be able to call genotypes accurately. We planned to obtain occurrence data from the global breeding and non-breeding range of the species from disparate sources, including national/regional/local surveys of breeding sites, individual satellite tracking data (for non-breeding occurrence), and verifiable citizen science records from biodiversity monitoring databases.
Data collection	Field data collected specifically for this study included blood samples, which were collected by the authors from breeding adults or nestlings. Occurrence data was partly collected by the authors and partly obtained from GBIF.org, eBird and surveys of breeding sites from several countries/regions, as detailed in Supplementary Table 7. Environmental data was derived from CHELSA, WorldClim, and Copernicus Global Land Service databases.
Timing and spatial scale	Blood samples were collected between 1993 and 2020 at 16 localities spread across the lesser kestrel global breeding distribution range. Sampling year is reported for each sample used in this study in Supplementary Data 1. Occurrence data were collected across the global breeding and non-breeding distribution of the lesser kestrel between 1978 and 2021, with details on locations of data collection and data sources reported in Supplementary Table 7.
Data exclusions	To improve the reliability of population structure analyses, we excluded 8 individuals from the ddRAD dataset and 2 individuals from the mtDNA dataset because they were related up to 3rd degree with at least another individual included in the dataset. The degree of relatedness of individuals in the sample is reported in Supplementary Data 1. Breeding and non-breeding occurrence records within the same 2.5 arc-minute grid cell (corresponding to the spatial resolution of climatic data) or duplicate records were pooled into a single occurrence record before fitting SDMs. Occurrence data used for fitting SDMs is reported in Supplementary Data 3.
Reproducibility	All methods used are thoroughly described to allow reproducibility. We provide the code to reproduce the analyses at https://doi.org/10.5281/zenodo.14988067
Randomization	As the study is observational, there was no need for random allocation of samples/individuals to groups. However, we relied on randomization procedures for conducting statistical analyses of both genetic and ecological data.

Blinding	Blinding was not relevant because the study design involved opportunistic sampling from individuals of a wild species from selected sampling localities. However, DNA sequencing was performed blind of the geographic origin of biological samples.

Did the study involve field work? ☒ Yes ☐ No

Field work, collection and transport

Field conditions	Fieldwork conducted specifically for this study involved the collection of blood samples only. Blood samples were collected at each sampling locality during the breeding season (June-July) from both adult birds and nestlings during excursions to breeding sites. There were no relevant field conditions to report.
Location	Coordinates of each sampling locality are reported in Supplementary Data 1.
Access & import/export	Specific authorizations for conducting field work at the different sampling localities were provided by the following local or national authorities (permit references in brackets): Croatian Ministry of Environment (KL: UP/I-612-07/15-48/108; URBROJ: 517-07-1-1-1-15-3; from 6 July 2015), Hellenic Ministry of Environment and Energy (ΒΕΝΔ0-ΔΔ8, ΒΛ9Σ0-Γ3Α, ΩΗΛΔ465ΓΘΗ-3ΓΙ), Israel Nature and Parks Authority (2015/40829), ISPRA (Law 157/1992 [Art.4 (1) and Art. 7 (5)]), Regione Sicilia (1616/2014), Ministry of Environment and Tourism of Mongolia (06/2564), Institute for Nature Conservation and Forests (ICNF) of Portugal (158/2013, 82/2020), Consejería de Medio Ambiente, Junta de Andalucía, Spain (SGYB-AFR-CMM), General Directorate of Nature Conservation and National Parks (MoAF) of Turkey (21264211-288.04-E.2059415). Tissue samples used for generating the reference genome were shipped to the USA under CITES permit n. IT/EX/2019/MCE/00336.
Disturbance	Disturbance to the breeding colonies was minimised by collecting blood samples from a small number of individuals at each sampling locality, following standard ethical practices and guidelines in studies of avian species. The amount of blood obtained for each individual was very small (approximately 50 microliters) compared to body size of both nestlings (>50 g) and adults (about 140 g) and there were no obvious effects of blood sampling on health state of sampled individuals. After sampling , which was performed with the minimum time required to handle every bird (mostly less than 10 min), birds were released back in their nests.

Reporting for specific materials, systems and methods

We require information from authors about some types of materials, experimental systems and methods used in many studies. Here, indicate whether each material, system or method listed is relevant to your study. If you are not sure if a list item applies to your research, read the appropriate section before selecting a response.

Materials & experimental systems

n/a	Involved in the study	
☒	☐	Antibodies
☒	☐	Eukaryotic cell lines
☒	☐	Palaeontology and archaeology
☐	☒	Animals and other organisms
☒	☐	Clinical data
☒	☐	Dual use research of concern
☒	☐	Plants

Methods

n/a	Involved in the study	
☒	☐	ChIP-seq
☒	☐	Flow cytometry
☒	☐	MRI-based neuroimaging

Animals and other research organisms

Policy information about studies involving animals; ARRIVE guidelines recommended for reporting animal research, and Sex and Gender in Research

Laboratory animals	The study did not involve laboratory animals.
Wild animals	Blood samples from lesser kestrels were collected in the field by capturing individuals on their nests at several localities across the species' breeding range between 1993 and 2020. Sex and age for each individual are reported in Supplementary Data 1.
Reporting on sex	Sex for each individual sampled for genetic analyses is reported in Supplementary Data 1. For adults, sex was determined in the field based on the highly sexually dimorphic colouration of the species, and subsequently genetically confirmed. For nestlings, it was determined based on sex-specific molecular markers. A few nestlings, included in mitogenomic analyses only, could not be sexed molecularly (n = 5). Regarding the sample of sexed individuals used for population genetic structure analyses (n = 84), both sexes were similarly represented (females: 45%, n = 38; males: 55%, n = 46). No sex-based analysis was performed as sex is not generally considered relevant for analyses of range-wide population structure.
Field-collected samples	Blood samples were collected and stored in screw-top vials containing 100% ethanol and kept at 4°C until DNA extraction, or collected using NucleoCards (Macherey-Nagel) and stored at room temperature.

Ethics oversight	All sampling conducted for this study complied with relevant regulations. Details of authorizations required for conducting fieldwork and sampling are reported in the Acknowledgements. No animal was euthanized or collected for this study.

Note that full information on the approval of the study protocol must also be provided in the manuscript.

Plants

Seed stocks	*Report on the source of all seed stocks or other plant material used. If applicable, state the seed stock centre and catalogue number. If plant specimens were collected from the field, describe the collection location, date and sampling procedures.*
Novel plant genotypes	*Describe the methods by which all novel plant genotypes were produced. This includes those generated by transgenic approaches, gene editing, chemical/radiation-based mutagenesis and hybridization. For transgenic lines, describe the transformation method, the number of independent lines analyzed and the generation upon which experiments were performed. For gene-edited lines, describe the editor used, the endogenous sequence targeted for editing, the targeting guide RNA sequence (if applicable) and how the editor was applied.*
Authentication	*Describe any authentication procedures for each seed stock used or novel genotype generated. Describe any experiments used to assess the effect of a mutation and, where applicable, how potential secondary effects (e.g. second site T-DNA insertions, mosiacism, off-target gene editing) were examined.*
